# Inside out: heart rate monitoring to advance the welfare and conservation of maned wolves (*Chrysocyon brachyurus*)

**DOI:** 10.1093/conphys/coab044

**Published:** 2021-06-24

**Authors:** Rosana N Moraes, Timothy G Laske, Peter Leimgruber, Jared A Stabach, Paul E Marinari, Megan M Horning, Noelle R Laske, Juan V Rodriguez, Ginger N Eye, Jessica E Kordell, Marissa Gonzalez, Tom Eyring, Christopher Lemons, Kelly E Helmick, Kristina M Delaski, Lisa H Ware, Julia C Jones, Nucharin Songsasen

**Affiliations:** 1 Center for Species Survival, Smithsonian Conservation Biology Institute, Front Royal, VA, 22630, USA; 2 Department of Physiology, Federal University of Parana, Curitiba, PR, 81530-900, Brazil; 3 Department of Surgery, University of Minnesota, Minneapolis, MN, 55455, USA; 4 AF Solutions, Medtronic Inc., Mounds View, MN, 55112, USA; 5 Conservation Ecology Center, Smithsonian Conservation Biology Institute, Front Royal, VA, 22630, USA; 6 Department of Parks and Recreation, Maryland-National Capital Park and Planning commission, Clinton, MD, 20735, USA; 7 Department of Conservation Medicine, Smithsonian Conservation Biology Institute, Front Royal, VA, 22630, USA

**Keywords:** Anthropogenic changes, autonomic nervous system, biologging, canids, cardiac physiology, stress

## Abstract

Anthropogenic change is a major threat to individual species and biodiversity. Yet the behavioral and physiological responses of animals to these changes remain understudied. This is due to the technological challenges in assessing these effects *in situ*. Using captive maned wolves (*Chrysocyon brachyurus*, *n* = 6) as a model, we deployed implantable biologgers and collected physiological data on heart rate (HR) and heart rate variability (HRV) over a 1-year period. To test for links between HR and changes in the environment we analysed HR daily rhythms and responses to potential stressors (e.g. physical restraint, change in housing conditions, short-distance transportation and unfamiliar human presence). The 2-min HR averages ranged from 33 to 250 bpm, with an overall rest average of 73 bpm and a maximum of 296 bpm. On average, HRV was higher in females (227 ± 51 ms) than in males (151 ± 51 ms). As expected, HR increased at dusk and night when animals were more active and in response to stressors. Sudden decreases in HR were observed during transportation in three wolves, suggestive of fear bradycardia. We provide the first non-anesthetic HR values for the species and confirm that behaviour does not always reflect the shifts in autonomic tone in response to perceived threats. Because strong HR responses often were not revealed by observable changes in behaviour, our findings suggest that the number and variety of stressors in *ex situ* or *in situ* environments for maned wolves and most wildlife species may be underestimated. Our study also shows that integrating biologging with behavioral observations can provide vital information to guide captive management. Similar technology can be used to advance *in situ* research for developing more effective welfare, management and conservation plans for the species.

## Teaser

Human-induced changes in the environment threaten the welfare and conservation of wildlife. By using the endangered maned wolf as a model, we show how implantable heart rate monitors can be used to study and monitor behavioral and physiological responses of animals to change. The results can be used to improve animal welfare and species conservation in captivity and the wild.

## Introduction

Rapidly expanding human populations are now affecting wildlife species globally (Bongaarts, 2019). Growing anthropogenic activities cause loss and fragmentation of natural habitat, leading to increased road mortality, reduced wildlife movements and population decline (Carvalho *et al.*, 2009; Abra *et al.*, 2018; de Souza *et al.*, 2018; Barbosa *et al.*, 2020). These direct threats often amplify conflict between wildlife and people and increase exposure of wildlife to domestic animals and zoonotic diseases (Paula and DeMatteo, 2015). Indirect effects from human-induced changes may be just as significant, although initially less obvious (Laske *et al.*, 2011; Støen *et al.*, 2015). As wildlife is gradually forced into stressful environments, such as agricultural landscapes and urban areas, species fitness and reproduction may decline (Jachowski *et al.*, 2012; Spercoski *et al.*, 2012; Vynne *et al.*, 2014). Therefore, expanding our understanding of the complex physiological (internal) and behavioral (external) responses to changing environments is an essential building block for a comprehensive, evidence-based approach to the assessment of wildlife welfare (Mellor *et al.*, 2015) and conservation (Jachowski and Singh, 2015).

Physiological adjustments to predictable changes, such as sexual maturation and seasonal fluctuation in ambient temperature, are part of an animal’s natural life cycle and represent evolutionary processes that aid in survival. Animals are also confronted with many unpredictable physical and social events (e.g. predator encounters), which require immediate physiological and behavioral responses (McEwen and Wingfield, 2010). These responses have evolved over time, generating complex physiological mechanisms of adaptation to stress in which different mediators actively deviate from basal levels in response to perceived stressors. These transient, stress-induced mechanisms promote stability or allostasis (‘achieving stability through change’) (Rao and Androulakis, 2019), by affecting signalling molecules such as glucocorticoids, which are considered beneficial to host survival by mobilizing energetic resources. However, when chronically elevated, glucocorticoids can disrupt adaptive mechanisms, limiting the individual’s ability to cope with new challenges (Rao and Androulakis, 2019).

The two main mediators of allostasis are the hypothalamus–pituitary–adrenal (HPA) axis and the sympathetic nervous system (SNS). In wildlife, the HPA axis activation in response to stress can be measured non-invasively by quantifying glucocorticoids or their metabolites in urine, faeces and hair (Heimbürge *et al.*, 2019; Palme, 2019). The resulting glucocorticoid measurements can be used to retrospectively assess physiological responses of animals to stressors and environmental changes. Yet, assessment of the SNS activation [e.g. increased heart rate (HR)], as a more direct and effective measure of neural activation in response to environmental stimuli, is still missing for most wildlife species. This is mainly due to the difficulty of collecting long-term physiological data without affecting the animal’s behaviour (Jachowski and Singh, 2015).

Advances in biologging technologies over the past decades have enabled the long-term collection of previously inaccessible physiological data, including measurements of HR (Laske *et al.*, 2018; Madliger *et al.*, 2018). Changes in HR and HR variability (HRV; the natural variation in time between heartbeats) reflect complex heart–brain interactions and autonomic nervous system dynamics (McCraty *et al.*, 2009), revealing information about the probable perception and processing of a given situation by an individual animal. For instance, free-ranging black bears encountering an unmanned aerial vehicle for the first time showed a spike in HR, followed by habituation after repeated exposures (Ditmer *et al.*, 2015; Ditmer *et al.*, 2018b). HR data also provided evidence that black bears (*Ursus americanus*) anticipate the risks of crossing roads, based on significant increases in HR before road-crossing events (Ditmer *et al.*, 2018a). Decreased HRV (indicating activation of the SNS) during the hunting season, or in close proximity to human settlements, strongly supports the concept of human-induced landscape of fear in brown bears (*Ursus arctos*) (Støen *et al.*, 2015). In Przewalski’s horses (*Equus przewalskii*), decreases in HRV along with increases in HR during spring have indicated an increase of allostatic load that is most likely associated with seasonal ‘stress’ (Pohlin *et al.*, 2017).

**Table 1:** Demographic information about maned wolves implanted with a biologger (Reveal LINQ™, Medtronic Inc., MN, USA) for a heart monitoring study at the Smithsonian Conservation Biology Institute, Front Royal, VA, from 2018 to 2019

Subject	Studbook number	Rearing method	Age (y)^*^	Body weight (kg)^*^	Implantation day
Female 1	3253	Parent	6.4	23.3	06/27/2018
Female 2	3438	Parent	3.5	24.3	06/27/2018
Female 3	3525	Parent	2.6	23.7	07/24/2018
Male 1	3524	Parent	2.5	24.3	06/26/2018
Male 2	2954	Hand	11.5	28.5	06/26/2018
Male 3	3531	Hand	2.5	24.5	07/24/2018

^*^At the time of implantation

Here, we present the results of the first study using an implantable biologger device for long-term physiological monitoring of HR in maned wolves (*Chrysocyon brachyurus***)**. The maned wolf is one of the most evolutionarily distinct species in the Canidae family (Perini *et al.*, 2010), with a total population currently estimated at only around 17 000 individuals in the wild (Paula and DeMatteo, 2015). It is considered a ‘keystone’ species and plays a major role in ecosystem dynamics, including seed dispersal and pest control (Consorte-McCrea, 2013). Over the past two decades, increased anthropogenic activities have caused drastic landscape changes to the maned wolf’s historical habitats in the Cerrado, Chaco and Pampas regions of South America (Queirolo *et al.*, 2011; Emmons, 2012; INPE, 2018). These changes include contraction of the southern limits of the species’ range (Queirolo *et al.*, 2011) and severe fragmentation (Carvalho *et al.*, 2009), resulting in isolated fragments of native ecosystems surrounded by farmlands. While maned wolves show some adaptability to human disturbance (Coelho *et al.*, 2008, 2018; Lyra-Jorge *et al.*, 2008; Carvalho *et al.*, 2009; Vynne *et al.*, 2011; Massara *et al.*, 2012; Péron *et al.*, 2017), there is evidence of higher stress levels in maned wolves living in altered landscapes than those living in natural habitats. Specifically, research has shown that faecal glucocorticoid metabolites concentrations increase with increasing distance from natural habitat patches and during the peak of harvest activity in croplands (Spercoski *et al.*, 2012; Vynne *et al.*, 2014). Other anthropogenic effects include higher exposure to pathogens from domestic animals (Curi *et al.*, 2012) and increased risks of roadkill (Barbosa *et al.*, 2020). In addition, the global *ex situ* population, which is kept as a safeguard against extinction of wild population, suffers from low reproductive success and sub-optimal health (Songsasen and Rodden, 2010; Jones *et al.*, 2018). For instance, a major factor in mortality in captive maned wolves is gastrointestinal diseases (Diniz *et al.*, 1999; Maia and Gouveia, 2002; Childs-Sanford and Angel, 2006), including inflammatory bowel disease (IBD; Padilla and Hilton, 2015). IBD is believed to result from complex interactions involving the immune system, the gut microbiome and genetic factors (Henson *et al.*, 2017; Bonaz *et al.*, 2018; Estruch *et al.*, 2020), with stress playing an important role in its pathophysiology through inhibition of the vagus nerve (Bonaz *et al.*, 2018; Payne *et al.*, 2019). Reduced vagal tone, as assessed by HRV, is associated with gut microbiota dysbiosis (imbalances in the composition and function of microbiome) and peripheric inflammation in humans and rodents (Bonaz *et al.*, 2018; Payne *et al.*, 2019). In maned wolves, autonomic imbalances due to potential environmental stressors together with a potential genetic predisposition to IBD (Henson *et al.*, 2017) may increase the risk of dysbiosis, gastrointestinal diseases and poor body condition in *ex situ* or *in situ* populations. The long-term effects of these health issues on the fitness of individuals and populations are yet to be elucidated (Acevedo-Whitehouse and Duffus, 2009) but would be helped by increased capacity for long-term physiological monitoring of heart parameters as a metric of autonomic balance.

Our objectives for this study were as follows: (i) evaluate whether implantable heart monitors can be successfully deployed in maned wolves; (ii) provide physiological HR parameters for the species; and (iii) generate case study examples of how HR monitoring can advance our understanding of individual welfare. Our main hypotheses were as follows: (i) HR in maned wolves increases with activity; and (ii) HR in maned wolves increases in response to stressful events (e.g. restraint, transportation and presence of unfamiliar people).

Our research provides the first baseline data on cardiovascular function in the maned wolf based on continuous measurements during daily activities. This goes well beyond routine veterinary assessments of HR and HRV during examinations when animals are regularly anesthetized. The resulting data, presented here, are unique and allow us to assess physiological responses to changes in the environment that may not be detectable by behavioral observations or other assessment methods.

## Materials and methods

### Subjects

Our study included six adult maned wolves (three males and three females) housed at the Smithsonian Conservation Biology Institute (SCBI), Front Royal, VA. Animals were either housed singly or in pairs, with or without offspring (Table 2 in Supplementary material—SM1), following the guidelines from the Association of Zoos and Aquariums’ Maned Wolf Species Survival Plan (AZA-MWSSP; Fletchall *et al.*, 1995). Individual animal age ranged from 2.4 to 11.5 years at the onset of the study. Mean body mass was 23.8 kg for females and 25.8 kg for males (Table 1). All individuals were provided a diet of custom maned wolf kibble (Mazuri, Land O’Lakes, Inc., Richmond, IN) with additional fruits (apple, tomatoes, papaya and banana), vegetables (lettuce), protein sources (mice, rats, fish and chicks) and *ad libitum* water. Study procedures were approved by the SCBI’s Institutional Animal Care and Use Committee (IACUC #18–22).

### Biologger implantation and data download

The maned wolves were implanted with the Reveal LINQ™ (Medtronic Inc., Minneapolis, MN) biologger, which was selected for its reduced dimensions (4.0 mm × 7.2 mm × 44.8 mm; mass, 2.4 g; volume, 1.2), long lifespan (up to 3 years of recording time), storage capacity (up to 400 d), wireless capability that enabled remote web-based monitoring and safety in other wildlife species (Laske *et al.*, 2018). To implant the biologger, wolves were anesthetized with a combination of ketamine (2 mg.kg^−1^), medetomidine (0.02 mg.kg^−1^) and midazolam (0.1 mg.kg^−1^) administered intramuscularly under squeeze crate restraint. Animals were intubated (11–12 mm ETT) and anaesthesia was maintained with isoflurane (0.5–1.5%). Meloxicam (0.2 mg/kg SQ) was administered for analgesia. At the end of the procedure, atipamezole was administered by intramuscular injection for an alpha-2-agonist reversal at five times the medetomidine administered. Using aseptic techniques, we made a small incision (~1 cm) in a left peristernal location over the heart area (~45 degrees on the sagittal plane). We then used the inserting tool packed with the biologger to open a subcutaneous pocket ~6 cm long, into which the device was injected. After verifying the optimal positioning by ensuring an acceptable R-wave amplitude (>0.15 mV) and appropriate rate detection, we closed the incision site using simple interrupted sutures (2–0 absorbable monofilament poliglecaprone 25 or braided polyglactin 910 suture) in the subcutaneous and skin layers. As procedures were performed in a controlled manner using aseptic technique and to reduce the risk of antimicrobial resistance to antibiotics (Vieira-da-Motta *et al.*, 2014), prophylactic antibiotics were not used. After implantation, animal care personnel visually inspected the animals twice daily for local tissue reaction at the implant site until full recovery.

Once implanted, we activated the biologger using transcutaneous telemetry (CareLink® Model 2090 Programmer) to record the time interval between each heartbeat and store average HR in beats per minute for every 2-min interval. This interval represents the current fixed setting of the custom software (‘B-Ware’; Laske *et al*., 2018) for use with the Reveal LINQ™ on animals. The biologger also saved waveform data (electrocardiograms—ECG) of the 10 most recent tachycardia and asystole episodes. Tachycardia was defined as HR ≥ 176 bpm sustained for at least 48 beats, based on ~70% of maximum HR values reported for domestic dogs of similar body mass (Noszczyk-Nowak *et al.*, 2009). Asystole was defined as at least 4.5 s between consecutive heartbeats according to proposed electrocardiographic classification for spontaneous syncope in humans and dogs (Brignole *et al.*, 2005; Perego *et al.*, 2020). The biologger software also calculated and stored daily HRV (standard deviation of beat-to-beat intervals in sinus rhythm using the average of 5-min segment medians during a 24-h recording; SDANN) and cumulative count of detected episodes (tachycardia or pauses). Also, the biologger’s internal accelerometer was programmed to record total activity every 15 min (number of active minutes). Animals were implanted between June and July 2018 and the data presented here include ~1 year of physiological data per individual.

**Figure 1:**
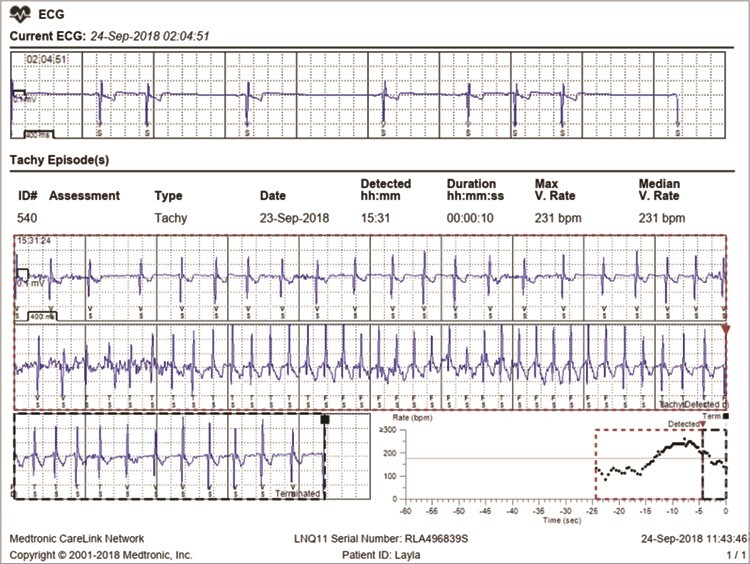
Example of a remote transmission from Female 2 to the Medtronic CareLink® Network containing (top) the 10-s strip of the current ECG on 24 September 2018 at 2:05 AM (HR = 54 bpm) and (bottom) the ECG of a tachycardia episode (#540) detected on the previous day at 3:31 PM (maximum HR of 231 bpm)

To download the various types of data stored by the biologger, we used remote transmission and direct telemetry. For remote transmissions, we mounted a monitor (My CareLink®, Medtronic Inc.) either inside or outside the dens, depending on the setting within the animal’s enclosure, but always within 2–3 m of the animals regular resting place. The biologger was programmed to connect wirelessly with its respective monitor every 2 h. To confirm the biologger’s sensing accuracy, we used a 10-s ECG strip at the time of transmission and the ECG associated with any tachycardia or asystole episode (Fig. 1). Both are recorded by the biologger and transmitted via the Global System for Mobile Communications to the individual repository created for data of each animal on the Medtronic CareLink® network. Direct telemetry downloads were done every two months when animals were physically held in a squeeze crate for the preventive control of ectoparasites or other medical procedures. For one of the hand-reared males (Male 2), who had previously been trained through positive reinforcement to remain close to the fence when called by animal care staff, we also attempted direct telemetry through the fence, without restraint. Data were retrieved by holding the telemetry head of the programming equipment (CareLink® Model 2090 Programmer) near the implant site (~5 cm telemetry range). Raw data (HR average every 2 min, daily HRV average and total minutes active every 15 min) were stored as Medtronic Programmer Data file for further analysis. To detect asynchrony between the internal clock of the biologger and the local time, we confirmed the time of the biologger in each data download.

### Responses to environmental stimuli

To identify specific environmental stimuli triggering HR responses, we collected information from the daily reports from animal care staff. This included the time animal care staff arrived and departed, identity and number of animal care staff members, unfamiliar human presence in the animal areas during maintenance activities (e.g. mowing or fence repair), physical restraint for preventive medicine and transportation. In addition, we used motion-activated trail cameras (DS4K trail camera, Stealth Cam®, TX, USA) to monitor animals’ activity. We mounted camera stations on the fence (two to four cameras per enclosure) and set them to continuously collect 10-s videos, with a 3-s delay between triggers. Camera stations for a breeding pair began in July 2018 and continued monitoring was undertaken for all individuals from October 2018 to August 2019. These videos were catalogued and used to identify behaviours displayed concurrently to tachycardia episodes (from the list of episodes for each animal on the Medtronic CareLink® network or from direct downloads). Also, we monitored animal reactions to implants using video recording by an observer. Video recording was conducted during the two weeks preceding and following implantation. Within 2 h before sunset, when animals are normally active and not disturbed by routine caretaking activities, observers positioned themselves either in a blind or in an observation tower. Observers allowed a 15-min acclimation period after arriving at the enclosure and recorded 10 min of video per animal per day. We used the Behavioral Observation Research Interactive Software (Friard and Gamba, 2016) to record the timing and duration of implant-related behaviours (examining, licking, scratching or gnawing at the implant site). Video annotations were completed by two observers who achieved 90% of agreement (Gamer *et al.*, 2019) at an initial analysis of 12 videos (~120 min).

To illustrate links between HR response and changes in the environment, we selected a few case examples from our catalogue of environmental stimuli that animals were exposed to during the study. Examples include husbandry or management procedures and social interactions commonly seen in captive environments.

Example 1 *—Physical restraint*: We assessed the HR response for the six wolves before, during and after restraint in a squeeze crate during data download (*n* = 19). Each restraint episode was divided into four periods, defined as relative minutes to the moment each subject entered the crate (time 0 min): *Baseline* (−60 to −40 min), *Pre*-*Crate* (−38 to −2 min), *In-Crate* (0 to 14 min) and *Post-Crate* (16 to 74 min). *Pre-Crate* included the arrival of animal care staff and researchers to the animal’s area ~40 min before the restraint. During *Pre-Crate* animals were coaxed by animal care staff (one or two persons) to enter an outdoor or indoor den, and then transferred from the den to the crate. When *In-Crate*, animals were squeezed to the side and one animal care staff held the telemetry head close to the implant site, while one to two researchers operated the equipment. Animal care personnel also classified the behavioral response of animals (*Calm* or *Stressed*) while *In-Crate*. During the *Post-Crate* period, animals were released into an area that included a den and an outside yard. All personnel left the area at the completion of the procedure. This sequence of events for capture and restraint is recommended by the Association of Zoos and Aquariums’ Maned Wolf Species Survival Plan (AZA-MWSSP) and routinely used at SCBI for management (e.g. transfers within the institution, monthly weighing) and medical procedures (e.g. vaccines, parasite control, delivery of anesthetic drugs).

 

Example 2 *—Change of enclosure and social grouping for Male 2*: At the onset of the study, Male 2 was housed in a family group, composed of the two parents and four 8-month-old offspring (two females and two males). Following the annual breeding recommendation from AZA-MWSSP, Male 2 was in the process of being relocated to a new enclosure. However, because we detected aggressive interactions between family members and changes on Male 2 trends for HR (increase) and HRV (decrease), the change of enclosure was expedited. Male 2 was separated from the family group in an adjacent yard and, after 5 days, moved to a new enclosure about 1000 m apart. After the move, Male 2 and Female 2 were housed singly in adjacent enclosures, separated by a double fence line, with visual, aural and olfactory contact. To test the effect of the intervention in reducing stress, we compared HR and HRV for Male 2 during two weeks before (including the 5 days in the adjacent yard) and after the move. We also provide a profile of the 2-min HR averages for Male 2 during the hours before and after the move.

 

Example 3 *—Short-range transportation by truck.* Three of the wolves (Female 2 and Males 2 and 3) were transported by truck inside SCBI for management purposes. On the transportation day, the three animals were restrained in a transportation box and held on the truck for ~14 min, including about 6 min of driving (distance of ~1000 m). During transportation, animal care personnel classified the animal’s behavioral response (*Calm* or *Stressed*). In this example, we plotted the 2-min HR averages for the visual inspection of the physiological response of animals to transportation.

### Statistical analysis of data

#### Responses to implants

We calculated the proportion of time animals displayed implant-related behaviours during the two weeks after implanting the biologger. Given that animals were not always visible to the observers (hidden inside dens or in the grass), the proportion values are relative to the total daily visible time per individual.

#### Biologger detection accuracy

To assess the biologger detection accuracy of heartbeats, we randomly selected 10-s ECG strips (30 per individual) by using the function *sort* in R (R Core Team, 2019) and counted the number of ventricular senses (VS) detected by the biologger (marked as VS in each ECG strip) and the number of VS identified by the visual analysis of each ECG strip (R waves). Detection error was calculated as the difference between the two values. Errors were classified as oversensing or undersensing if positive or negative, respectively. The causes of the errors were identified from the biologger VS marks on the ECG strips (i.e. T or P wave detection, muscular or electrical noise, signal drop).

#### Biologger data

Prior to analysis, we used the differences between the time of the biologger and local time over the 1-year period (mean absolute error = 22 min) to predict the correct date and time for each data point with individual linear regressions (R^2^ ~ 0.99). Then, we generated subsets of data using different time scales and grouping factors (e.g. subject, sex, light period, activity level). Time scales for HR subsets were 2-min averages, hourly average (mean of 30 recordings of 2-min HR averages) and daily average (mean of 720 recordings of 2-min HR averages). To calculate averages by light period, we first used the R packages *rgeos* (Bivand and Rundel, 2020) and *maptools* (Bivand *et al.*, 2020) to calculate the daily hours of sunrise, sunset and nautical dawn and dusk for the geographic coordinates of SCBI (−78.1395, 38.89292). Next, the corrected date and time for each data point of HR and activity were compared to the daily light periods and classified as *Dawn* (03 h:34 m to 06 h:28 m), *Dusk* (17 h:53 m to 20 h:54 m), *Day* (06 h:28 m to 17 h:53 m) or *Night* (20 h:54 to 03 h:34 m). Light period and subject were then used as grouping factors to generate daily averages of HR per light period. Daily individual activity data (ACT; active min per 15-min interval) were also grouped as activity per light period (sum of all ACT per light period). To assess how animals’ activity influenced HR, we used the time intervals from the ACT to calculate a 15-min HR average (alternating average of seven or eight consecutive 2-min HR averages recordings), aligned with the ACT time interval. Next, we calculated Spearman correlation coefficients between HR and ACT averages every 15 min. Also, we selected all non-active 15-min time intervals (ACT = 0) to calculate individual and overall HR ‘rest’ averages.

To test the differences among individuals in HR and HRV, we fit univariate linear mixed models with subject as the random effect to account for repeated measurements. To assess the effect of sex (*Male* or *Female*), rearing method (*Parent* or *Hand*), light period (*Day*, *Dusk*, *Night* or *Dawn*), restraint period (*Baseline*, *Pre*-*Crate*, *In*-*Crate* or *Post*-*Crate*), apparent behaviour in crate (*Calm* or *Stressed*) and number of previous restraint events for data download (*0* to *5*) on HR and ACT, we fit several linear mixed-effects models, with different structures and combinations of covariates. Since the categorical variables *Sex* and *Rearing Method* were correlated, they were not included simultaneously in any of the models. The best fit models were selected using Akaike’s information criteria and are summarized in Table 3 in Supplementary material—SM1. We included random effects in all models to account for repeated measurements within subjects and dates. Models were fit using the *lme4* R-package (Bates *et al.*, 2015). The assumptions of normality and homogeneity of the residuals and model stability were tested using the packages *sjPlot* (Lüdecke, 2020) and *performance* (Lüdecke *et al.*, 2020). Detailed output tables for all models are provided as supplementary material (Supplementary material—SM1).

To explore and test how HR and HRV in Male 2 were affected by the change of enclosure (Example 2), we used a Bayesian framework. Specifically, we used a Markov chain Monte Carlo (MCMC) simulation to analyse daily HR and HRV time-series data, spanning a 31-day period (15 days before and 15 days after the animals were moved). All MCMC modelling was performed with the *CausalImpact* package in R (Brodersen *et al.*, 2015). As a control time series, we used data for the same period from Male 1, which was housed with Female 1 and two pups in the same set of enclosures as Male 2 (prior to the move). Pre-intervention data (relative day −15 to day 0) were included in the model training. The post-intervention period (relative day 1 to day 15) was used for computing a counterfactual prediction. The causal effect of the change of enclosure was estimated as the difference between the observed time series and a hypothetical time series without change (Bayesian one-sided tail-area *P* value).

## Results

### Biologger implantation and data download

The total time between induction and reversal of anaesthesia ranged from 61 to 114 min with ~20–30 min for the biologger implantation procedure. Local tissue reaction to the implant was minimal and healing uneventful for most wolves.

Post-operative veterinary intervention was only required for Female 2, who received antibiotics and anti-inflammatory medication for incision site drainage and swelling. Within 1 year of implantation, all biologgers were fully functional with no significant subcutaneous migration or expelling.

During the first two weeks after the procedure, all individuals displayed certain implant-related behaviours, including licking, examining or gnawing at the implant area. On average, animals were engaged in these implant-related behaviours for 3.5% (ranging from 0.4% to 52.4%) of the visible time (Table 4 in Supplementary material—SM1). Licking of the incision site was the most prevalent implant-related behaviour (85%).

Data have been successfully downloaded using direct telemetry and remote transmission. Over the study period, we performed 38 direct data downloads, of which 36 (95%) were performed while the animals were held in a squeeze crate. The remaining data downloads (5%) were performed on an unrestrained male (Male 2) through the fence line. The procedure took on average 7 ± 2 min (range, 4–12 min), including downloading data, saving files and verifying software/system. The total download time was mainly affected by the number of interruptions in the telemetry signal caused by animals moving away from the telemetry head. We also received reports almost daily through the remote transmission system for some individuals. Frequency of remote transmissions depended on how often animals used the dens and the strength of the signal of the mobile telephone network in each area. Female 3 had the lowest transmission rate with an average of 5.8 transmissions per month, while Male 3 had the highest average (137.5 transmissions per month). ECG details of 1480 tachycardia episodes were transmitted for the six wolves out of a total cumulative count of ~8600 tachycardia episodes during the 1-year period.

Based on the visual evaluation of 180 randomly selected 10-s ECG strips (30 per animal), the number of VS (R waves) detected by the biologger was 100% accurate in 138 strips (77%). Failure to sense was detected in 23% of the ECG, either as oversensing (15%) or undersensing (8%). The highest rate of the sensing failures occurred for Female 3 (19 out of 30 ECG) and was caused mainly by noise (muscular or electrical) or T wave detection (high voltage T waves). For the remaining individuals, T wave detection and signal dropout were the main reasons for oversensing and undersensing, respectively. Differences between the number of VS confirmed by ECG analysis and the number of VS recorded by the biologger ranged from −9 to 11, with a mean absolute error of 0.8 VS per 10-s ECG.

### Heart rate parameters

**Figure 2:**
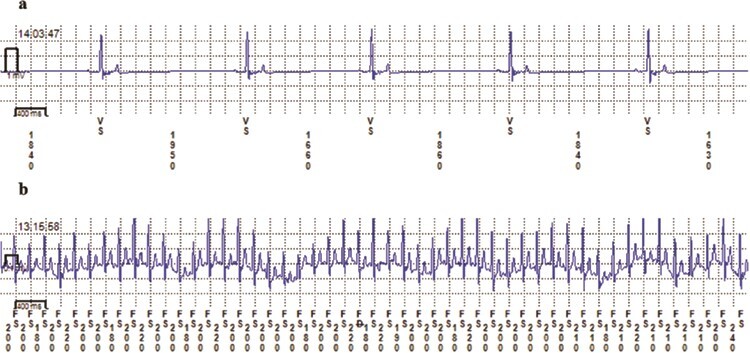
Remote transmissions sent to the Medtronic CareLink® Network from two captive maned wolves implanted with a biologger (Reveal LINQ™, Medtronic Inc., MN, USA). The 10-s ECG strips show (a) the minimum (33 bpm; Male 3) and (b) maximum (296 bpm; Female 3) HR values confirmed by ECG. Note: On the bottom marker channel, numbers represent the interbeat interval (ms) and letters are the sensing marks for ventricular sense (VS) and fast ventricular sense (FS)

**Figure 3:**
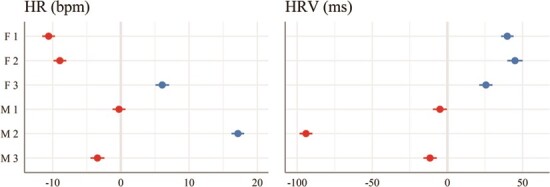
Best linear unbiased predictions (BLUPs; dots) and confidence intervals (95% CI; horizontal lines) from intercept-only linear mixed models (REML) to predict daily heart rate (HR) average and daily heart rate variability (HRV; SDANN) with individual maned wolf (3 Males and 3 Females) as random effect. The model’s intercept is at 89.7 [95% CI (81.4, 97.9); t(2045) = 21.19; *P* < 0.001] for HR and at 190.9 [95% CI (149.6, 232.3); t(1970) = 9.06; *P* < 0.001] for HRV. Blue and red dots represent prediction values, respectively, above or below the intercept (vertical grey line at value 0). Summaries for both models are provided (Models 1 and 2 in Supplementary material—SM1)

HR values range was wide and varied slightly based on the mode of data transmission. Among all individuals, data obtained via direct download revealed that 2-min HR averages ranged from 33 to 255 bpm (Fig. 1 in Supplementary material—SM2). However, based on the R-R intervals (interbeat interval) confirmed by remotely transmitted ECGs, the minimum and maximum HR values were 33 (mean R-R interval of 1788 ms; Fig. 2a) and 296 bpm (mean R-R interval of 202 ms; Fig. 2b), respectively. The effect of sex on daily HR average was not significant [95% confidence interval (CI), (−7.34, 25.30); t(2044) = 1.08; *P* = 0.281], but between-subject variation explained 57% of the variation in daily HR average (Fig. 3; Models 1 and 3 in Supplementary material—SM1).

Individual daily HRV average ranged from 97 ± 10 ms (Male 2) to 236 ± 47 ms (Female 2), with an overall average of 227 ± 51 ms in females and 151 ± 51 ms in males (Fig. 2 in Supplementary material—SM2). HRV varied significantly between males and females, with males having lower HRV. The effect of sex *Male* was significantly negative [beta = −73.8; 95% CI (−131.3, −16.4); t(1969) = −2.52, *P* < 0.05] and between-subject variation explained 61% of the total variance in daily HRV (Fig. 3; Models 2 and 4 in Supplementary material—SM1).

HR was correlated with animal activity and the Spearman correlation coefficient between HR average and total of minutes active every 15 min was 0.69 (*t* = 419, *df* = 197 099, *P* < 0.0001). The average HR during resting (ACT = 0) ranged from 63 (Female 1) to 93 bpm (Male 2), with an overall mean of 73 ± 17 bpm. When animals were active for all 15 min (ACT = 15), the HR average was 112 ± 22 bpm (min = 95; max = 130).

**Figure 4:**
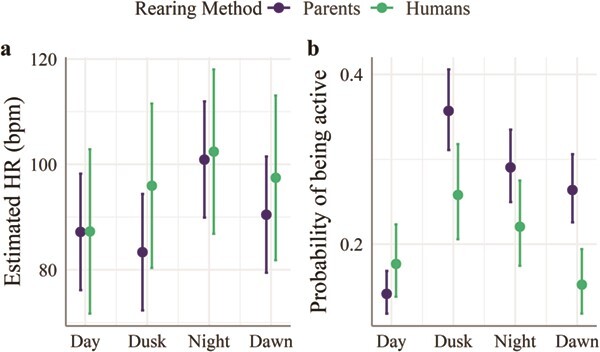
Estimated values for heart rate average (HR; a) and probability of being active (b) by daily light period and rearing method in six captive maned wolves at the Smithsonian Conservation Biology Institute (38°53′15.6″N 78°9′54.6″W), Front Royal, VA, USA. Within groups, all differences in relation to *Day* were significant (*P* < 0.001). The interaction effect of rearing method was significant (*P* < 0.001). The models’ intercepts, corresponding to Light = *Day* and Rearing Method = *Parent*, are at 87.2 [95% CI (76.2, 98.2); t(7029); *P* < 0.001] for HR (bpm) and at 0.16 [95% CI (0.12, 0.20), *P* < 0.001] for probability of being active (Models 5 and 6 in Supplementary material—SM1)

HR and ACT changed with daily light period, with a significant interaction of rearing method (Models 5 and 6 in Supplementary material—SM1). The estimated HR for wolves reared by parents was lowest during the day (81 bpm) and highest at dusk (103 bpm; Fig. 4a). Hand-reared wolves had a similar estimate for HR at dusk but the lowest HR estimate at dawn (87 bpm). Because hand-reared wolves had a high HR during the day (96 bpm), the estimated differences from day to dusk and night were smaller than in wolves reared by parents (Fig. 4a).

Along with HR trends, the probability of being active was lower during the day and at dawn, respectively, for wolves raised by parents and wolves raised by humans. Except for the day, hand-reared wolves had a lower probability of being active than parent-reared counterparts (Fig. 4b).

### Heart rate response to changes in the environment

**Figure 5:**
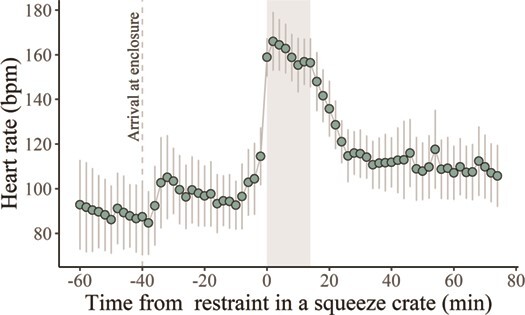
Heart rate averages every 2 min (bpm) in six captive maned wolves from 1 h before to 1 h after physical restraint (*n* = 19) in a squeeze crate (grey rectangle) for biologger data download. The dashed line indicates the time researchers and animal care staff arrived at the animal area

Example 1
*—Physical restraint*. Study animals showed strong HR responses to the physical restraint in a squeeze crate (Fig. 5). The overall HR average before the arrival of animal care staff and researchers to the animal area (~40 min prior to download; *Baseline*) was 89 ± 32 bpm. The impact of each restraint period (*Pre-Crate*, *In-crate* and *Post-Crate*) on HR was significantly affected by rearing method (Model 7 in Supplementary material—SM1). HR average for parent-reared wolves was higher than baseline in all restraint periods, with a peak when inside the crate (165 ± 20 bpm) higher than the peak for hand-reared wolves (141 ± 18 bpm). The lowest HR average during restraint was observed in Male 3 (129 ± 15 bpm) and the highest (179 ± 15 bpm) in Female 2.

During restraint, behaviour inside the crate (*Calm* or *Stressed*) did not predict HR response, independent of the rearing method (Model 8 in Supplementary material—SM1). The estimated HR averages for parent-reared wolves were 164 bpm and 171 bpm when they seemed *Calm* or *Stressed*, respectively. Hand-reared wolves’ estimates of HR average when *Calm* or *Stressed* were around 150 bpm, with a lower HR than parent-reared wolves when *Stressed*. In addition, the number of previous restraint events for data download only (*0* to *5*) did not predict the HR average *In-Crate* (Model 8 in Supplementary material—SM1).

Example 2 *—Change of enclosure and social grouping for Male 2*: HR and HRV were influenced by the relocation of Male 2, whose social environment changed from a family group to individual housing in a new enclosure. The relocation itself was stressful but daily HR average for Male 2 decreased from 108 ± 4 bpm to 95 ± 7 bpm after the move, approaching his resting HR average over the 1-year study period (93 bpm).

**Figure 6:**
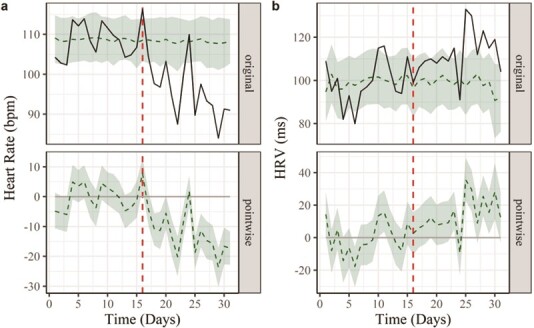
Effects of change in housing condition on average daily heart rate (HR) and heart rate variability (HRV) for a male maned wolf (Male 2). Male 2 was housed in a family group until day 16 and then moved (vertical red dashed line) to be singly housed. Plots are based on casual impact analysis. The solid lines in the top panels represent the original data and the shaded areas demonstrate the counterfactual prediction (95% confidence interval) for the intervention period (days 17 to 31), based on the trend calculation of the pre-intervention period (days 1 to 16). The dashed lines in the bottom panel are the point-wise difference between the original data and the counterfactual prediction. The change of housing conditions decreased HR (a; bottom panel) by 12% (CI: −14%, −9%; *P* = 0.001) and increased HRV (b; bottom panel) by 14% (CI: 10%, 19%; *P* = 0.002)

The change in daily HR in Male 2 was significant, as confirmed by causal impact analysis (Fig. 6a). The counterfactual prediction for daily HR in the absence of an intervention was 108 bpm (95% CI: 106, 111; Fig. 6a top panel), with an estimated relative causal effect of −12% (95% CI: −14%, −9%; Bayesian one-sided tail-area probability: *P* = 0.001; Fig. 6a bottom panel). Daily HRV (SDANN), on the other hand, increased from an average (± SD) of 99 ± 11 ms to 113 ± 10 ms, when comparing the 15 days prior and after the move, respectively (Fig. 6b, top panel). Casual impact analyses also detected a significant effect of the intervention on HRV, with an increase of 14% (95% CI: 10%, 19%; Bayesian one-sided tail-area probability: *P* = 0.002; Fig. 6b, bottom panel). Aggressive interactions along the fence line were detected on day 24 (Supplementary video, SM3), resulting in an HR spike (Fig. 6a, top panel) and a decline in HRV (Fig. 6b, top panel).

**Figure 7:**
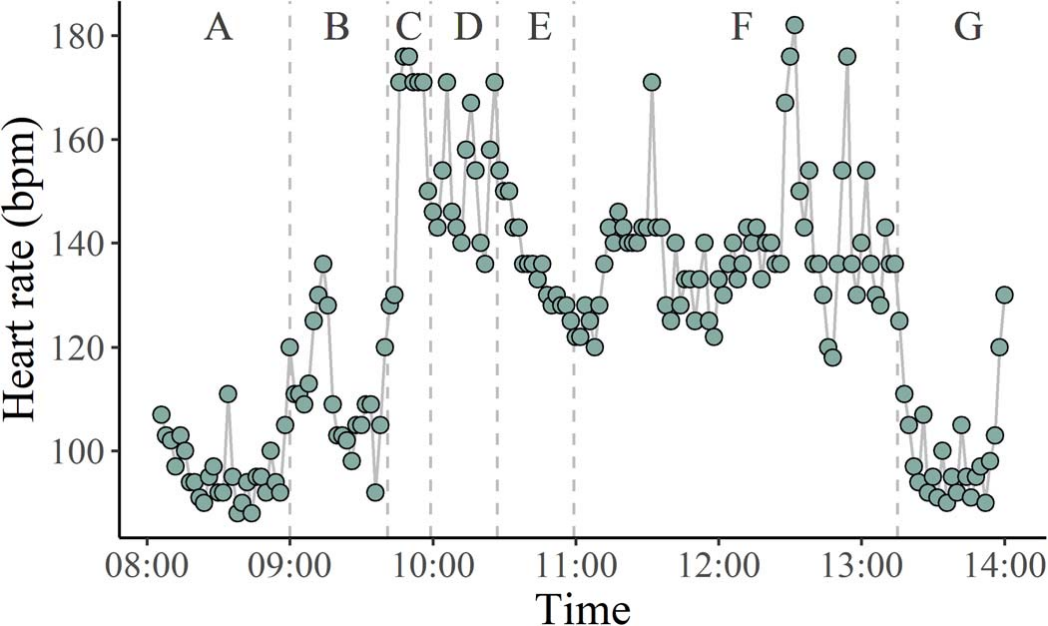
Heart rate averages every 2 min (bpm) for a male maned wolf (Male 2), during and after transfer to a new enclosure. Vertical lines indicated the start time for each event including: A = prior to animal care staff arrival; B = capture and restraint in a transportation crate; C = transportation by truck; D = transfer to a squeeze crate for a veterinary examination and biologger data download; E = released into the inner den; F = released into the outer area of the new enclosure; G = held inside the inner den

When looking into the detailed profile of the HR response on the day of the move for Male 2 (Fig. 7), the largest increase in the 2-min HR average (from 92 to 176 bpm) was observed during transportation by truck (Fig. 7C) to the new enclosure (~14 min long). Yet, while freely exploring the outer area of the new enclosure, Male 2 had initial contact (visual, aural, olfactory) with Female 2 in the adjacent yard, resulting in a sustained increase in HR (HR max = 182 bpm; Fig. 7F). HR averages returned to pre-transportation levels only when Male 2 was back to the inner den (Fig. 7G).

**Figure 8:**
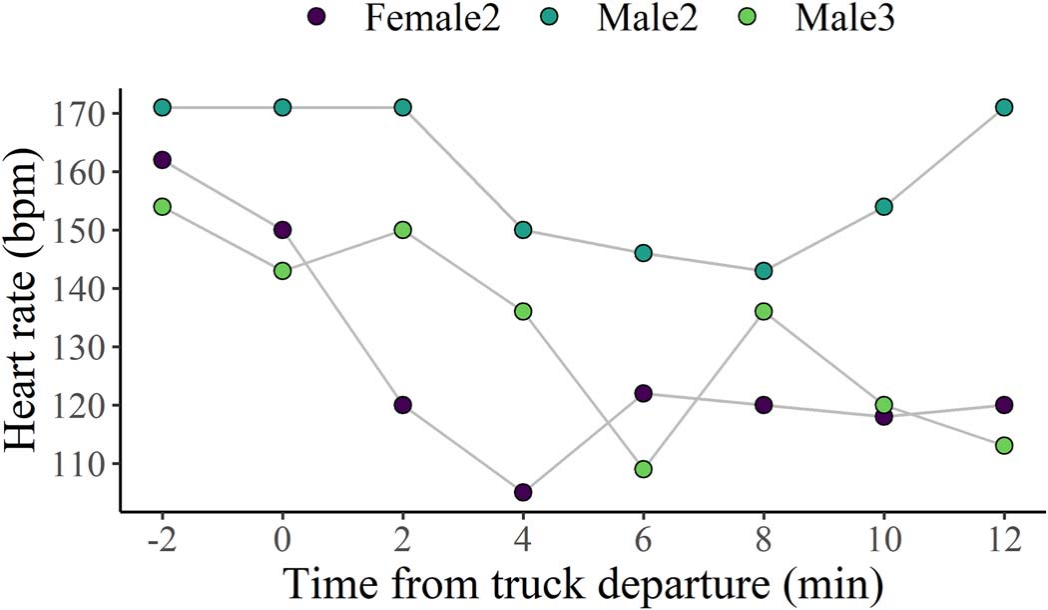
Heart rate averages every 2 min (bpm) for Female 2 and Males 2 and 3, during a short distance (~1000 m) transportation by truck, in a transportation box. Total time on the truck was ~14 min, with about 6 min driving (start = time 0)

Example 3 *—Short-range transportation by truck.* During the short-term transportation by truck, Female 2 and Males 2 and 3 all showed a bradycardic response, while behaving calmly, in a ‘frozen’ posture. The initial response to being loaded into the truck was an increase in HR, with a spike of 176 bpm for Male 2, 162 bpm for Female 2 and 154 bpm for Male 3. However, within 4–8 min after departure, HR decreased to 146 bpm for Male 2, 105 bpm for Female 2 and 109 bpm for Male 3 (Fig. 8).

## Discussion

In our study, we successfully deployed an implantable biologger into captive maned wolves and used high temporal resolution data sampling (HR averages every 2 min and ECG strips for tachycardia events) to monitor HR over a 1-year period. The surgical procedure is safe, with minimal impact on the health (local inflammation of the implant site) and behaviour (primarily licking the implant site) of the animals during healing. For the first time, we documented the species’ natural cardiac parameters and identified causal relationships between changes in HR and specific environmental factors triggering the ANS activation. Being able to identify the links between specific environmental parameters and autonomic nervous system (ANS) activation could advance the welfare of the *ex situ* population and leverage our understanding of human impact on wild wolves.

Our analysis of 2-min averages (bpm) and instantaneous HR (R-R intervals) revealed a wide natural range for the species, with differences up to 300 beats between minimum and maximum HR for the same individual (from 31 to 333 bpm). Data on cardiac parameters that are not altered by chemical or physical restraint in wild canids are limited or not available (Kreeger *et al.*, 1989, 1990a). However, these parameters were generally assumed to be similar to those of domestic dogs of comparable size (Larsen and Kreeger, 2014; Padilla and Hilton, 2015). The range of HR 2-min averages (31 to 255 bpm) in maned wolves observed in the present study is similar to those reported for grey wolves (Kreeger *et al.*, 1990a) and domestic dogs (Noszczyk-Nowak *et al.*, 2009). Yet, HR ranges reported previously for anesthetized maned wolves (from 113 to 178 bpm; Furtado *et al.*, 2006; Estrada *et al.*, 2009; Dias *et al.*, 2015) exceed the resting HR average in our study (73 bpm). This difference is probably due to the impact of anaesthesia on cardiac function. For example, a study in grey wolves has shown that HR of anesthetized wolves is higher than those observed without chemical restraint (Kreeger *et al.*, 1990b).

Low HR values around 30 bpm were recorded during sleep for Male 3 (Fig. 2a), suggesting an increase in parasympathetic tone during sleep in maned wolves, as reported for other mammals (Viola *et al.*, 2002). High HR values, by contrast, are primarily a consequence of increased SNS activity during physical or psychological stress (Viola *et al.*, 2002). The maximum HR (2-min average) recorded in our study (296 bpm; Fig. 2b) is higher than that reported for dogs of similar size during maximal physical activity (276 bpm; Taylor *et al.*, 1987). The maximum instantaneous HR (R-R interval) for the same ECG (Fig. 2b) is even higher (333 bpm or an interbeat interval of 180 ms), approaching HR values reported for red foxes, when they run or are chased by humans (Kreeger *et al.*, 1989). The cause for the HR increase seen in the ECG for Female 3 (Fig. 2b) was the use of a lawn mower in the enclosure and the noise from a nearby construction site, suggesting a psychological stress event. In addition, while the detection error was minimal and likely did not affect the 2-min HR averages, biologger sensing error may contribute to greater individual HR and HRV variation, as we found for Female 3 (Fig. 1, SM2). Access to stored/transmitted ECGs was therefore key to our study to confirm the accuracy of the data.

Differences in the social contexts and individual’s personality can result in significant variations in HR and HRV responses to changes in the environment (Kovács *et al.*, 2015; Finkemeier *et al.*, 2019). Although unable to test the impact of personality or social housing on HR in our study, anecdotal evidence from our animal care personnel suggests that these two factors may have contributed to the observed variability among individuals. In addition, daily changes in light and animal’s activity partially explained the variation in HR, with higher HR averages observed at dusk and night, when animals were more active. Rearing method affected the daily activity pattern in the present study, as described in other zoo animals (Berger, 2011; Yerga *et al.*, 2015), with hand-reared wolves being less ‘nocturnal’ than parent-reared wolves. Yet, this outcome should be confirmed with a larger sample size. The HR values reported here for captive animals may differ from wild maned wolves due to differences in daily activity and environmental temperature (Constable *et al.*, 1998). More specifically, animals in our study were on average less active (7 ± 3 h a day) than free-ranging maned wolves (10 to 16 h a day; Emmons, 2012), and exposed to lower annual temperature averages (6–19°C; Lawrimore *et al.*, 2016) than those of the Brazilian Cerrado (18–27°C; Silva *et al.*, 2008).

HR responses to common captive stressors in our study, such as physical restraint and forced proximity to humans or conspecifics, may be interpreted as predator-induced fear experiences (Zanette *et al.*, 2019), which induces a typical arousal response, with activation of the sympathetic (increased HR and blood pressure) and somatomotor (increased muscular tonus) nervous system (Kozlowska *et al.*, 2015). For instance, restraint for data downloads has resulted in a spike in HR in all individuals (Fig. 6), regardless of behavioral response (*Calm versus Stressed*) inside the crate. Being hand-reared attenuated the overall HR response to restraint, probably because the animals are accustomed to human presence (Carlstead, 2009). However, repeated restraint events for downloading data did not cause habituation, which could reflect a lasting fear memory to restraint and unfamiliar human presence, similar to the predator-cue fear memory recently demonstrated in the behaviour and brain of a wild animal (Zanette *et al.*, 2019).

In this study, maned wolves also displayed decreases in HR during stressful events, which could be the first documentation of ‘fear bradycardia’ (Kreeger *et al.*, 1990a; Stemmler *et al.*, 2007; Kozlowska *et al.*, 2015) for the species. Physiologically, when animals are prevented from escaping when they experience fear (e.g. restraint), arousal can be followed by ‘freezing’ of flight response and parasympathetic activation (Kozlowska *et al.*, 2015). Vagal nerve stimulation will then attenuate HR increase to fear or decrease HR shortly after the animal is captured. In our study, Female 2 and Males 2 and 3 showed a bradycardic response during short-range transportation, while remained in a ‘frozen’ posture. Rather than fear bradycardia, a decrease in HR could be an indication of the animal’s habituation to the situation. However, this is unlikely in our examples because capture and transportation are identified as very stressful for wildlife and domestic animals (Dickens *et al.*, 2010).

An additional source of stress identified in our study was the forced cohabitation within the family group for Male 2 (Fig. 6). In maned wolves the social system is dispersed and animals are mostly solitary, although parents share the responsibility in offspring care (Dietz, 1984; Bandeira de Melo *et al.*, 2007; Emmons, 2012; Péron *et al.*, 2017). The spacing of individuals is based on signals such as long-range vocalizations, visual threat displays and scent marking to promote avoidance (Kleiman, 1972). In confined environments, despite efforts to provide ideal husbandry conditions, the natural social structure cannot be easily maintained, often resulting in stressful conspecific interactions. The inevitable proximity to conspecifics in captivity could cause sustained stress as indicated by the increase in daily HR average and the reduction of HRV for Male 2. This confirms earlier findings of co-housed related females, which have shown increased faecal glucocorticoid metabolites as well as sex steroids suppression (Jones *et al.*, 2018). Isolation of Male 2 from the group and relocation to a new enclosure resulted in a sustained decrease in HR and an increase in HRV, suggesting overcrowding was responsible for SNS activation in this male. HR data collected on a fine time scale were essential to evaluate the impact of each step during relocation. Initially, the displacement itself induced activation of the SNS, with spikes in the HR as Male 2 explored the new environment (new enclosure) and established boundaries with a neighbouring female (Female 2). During these initial hours at the new enclosure, HR increases (Fig. 7F) were similar to or higher than those of transportation (Fig. 7C) or restraint (Fig. 7D). Later, during the introductions for the breeding season, Male 2 and Female 2 were allowed contact through a protected barrier, when we observed initial aggressive interactions, as shown in the example we captured with one of our trail cameras (Supplementary material—SM3). These interactions resulted in spikes in HR and increases in HR daily average in both individuals.

In conclusion, we demonstrated that a miniaturized implantable biologger can be successfully used for the long-term monitoring of HR in captive maned wolves. The same protocols can potentially be applied to most carnivores of similar or smaller size after risk assessment (Casper, 2009). This new methodological approach provided the first metrics of the natural heart rhythms for the species and, when integrated with behavioral assessment and animal care reports, revealed that animals’ internal (activation of SNS) and external responses (behaviour) do not always match. Individuals seen as behaviourally calm might be experiencing a strong SNS activation in response to a perceived threat, suggesting that the sources of stress in *ex situ* or *in situ* environments might be underestimated for maned wolves and most other wildlife species. The association between decreased parasympathetic tone and common diseases found in captive or wild maned wolves is yet to be determined. However, the negative effects of stress responses to erratic environments can exceed the mechanisms of counteraction or mitigation, exerting additional pressure on the function of the immune system and ultimately compromising the health of *ex situ* and *in situ* populations (Acevedo-Whitehouse and Duffus, 2009). Despite our small sample size, with two of the individuals being hand-reared, we provided strong evidence of the potential direct applications of HR monitoring for captive management. We also demonstrated that heart rate monitoring, especially when combined with hormonal, behavioral and ecological metrics, has a tremendous potential as a tool to assess the impact of environmental change on species health and fitness of wildlife living *in situ*. Yet, for this technology to be routinely utilized in *in situ* settings, there is still a need for developing new technologies that will allow remote data download to avoid repeated capture and restraint.

## Supplementary Material

suppl_data_coab044

## References

[ref1] Abra FD, Huijser MP, Pereira CS, Ferraz KMPMB (2018) How reliable are your data? Verifying species identification of road-killed mammals recorded by road maintenance personnel in São Paulo State, Brazil. Biol Conserv 225: 42–52.

[ref2] Acevedo-Whitehouse K, Duffus ALJ (2009) Effects of environmental change on wildlife health. Philos Trans R Soc B Biol Sci. 364: 3429–3438. 10.1098/rstb.2009.0128 PMC2781848 19833653

[ref3] Bandeira de Melo LF, Lima Sábato MA, Vaz Magni EM, Young RJ, Coelho CM (2007) Secret lives of maned wolves (*Chrysocyon brachyurus* Illiger 1815): as revealed by GPS tracking collars. J Zool 271: 27–36.

[ref4] Barbosa P, Schumaker NH, Brandon KR, Bager A, Grilo C (2020) Simulating the consequences of roads for wildlife population dynamics. Landsc Urban Plan 193. doi: 10.1016/j.landurbplan.2019.103672. PMC6961961 31942086

[ref5] Bates D, Mächler M, Bolker BM, Walker SC (2015) Fitting linear mixed-effects models using lme4. J Stat Softw 67: 1–48.

[ref6] Berger A (2011) Activity patterns, chronobiology and the assessment of stress and welfare in zoo and wild animals. Int Zoo Yearb 45: 80–90.

[ref7] Bivand R, Lewin-Koh N, Pebesma E, Archer E, Baddeley A, Bearman N, Bibiko H-J, Brey S, Callahan J, Carrillo G, *et al.* (2020) maptools: Tools for Handling Spatial Objects. R package version 1.0-2. R Package version 10-2.

[ref8] Bivand R, Rundel C (2020) Interface to Geometry Engine—Open Source (GEOS): Package “rgeos”. In R package version 0.5-5. *R Doc*.

[ref9] Bonaz B, Bazin T, Pellissier S (2018) The vagus nerve at the interface of the microbiota–gut–brain axis. Front Neurosci. 12: 49. 29467611 10.3389/fnins.2018.00049 PMC5808284

[ref10] Bongaarts J (2019) IPBES, 2019. Summary for policymakers of the global assessment report on biodiversity and ecosystem services of the Intergovernmental Science-Policy Platform on Biodiversity and Ecosystem Services. Popul Dev Rev 45: 680–681.

[ref11] Brignole M, Moya A, Menozzi C, Garcia-Civera R, Sutton R, Garciacivera R, Sutton R (2005) Proposed electrocardiographic classification of spontaneous syncope documented by an implantable loop recorder. Europace 7: 14–18. 15670961 10.1016/j.eupc.2004.11.001

[ref12] Brodersen KH, Gallusser F, Koehler J, Remy N, Scott SL (2015) A comparative approach to the study of Keeper-Animal Relationships in the zoo. Zoo Biol 28: 589–608. 10.1002/zoo.20289 19885915

[ref85] Carlstead K (2009) A comparative approach to the study of Keeper-Animal Relationships in the zoo. In A Consorte-McCrea, E Santos, eds, Ecology and Conservation of the Maned Wolf: Multidisciplinary Perspectives. CRC Press, Boca Raton, pp. 35–52.

[ref13] Carvalho FMVV, De Marco P, Ferreira LG (2009) The Cerrado into-pieces: habitat fragmentation as a function of landscape use in the savannas of central Brazil. Biol Conserv 142: 1392–1403.

[ref14] Casper RM (2009) Guidelines for the instrumentation of wild birds and mammals. Anim Behav 78: 1477–1483.

[ref15] Childs-Sanford SE, Angel CR (2006) Transit time and digestibility of two experimental diets in the maned wolf (*Chrysocyon brachyurus*) and domestic dog (*Canis lupus*). Zoo Biol 25: 369–381.

[ref16] Coelho CM, De Melo LFB, Sábato MAL, Vaz Magni EM, Hirsch A, Young RJ (2008) Habitat use by wild maned wolves (*Chrysocyon brachyurus*) in a transition zone environment. J Mammal 89: 97–104.

[ref17] Coelho L, Romero D, Queirolo D, Guerrero JC (2018) Understanding factors affecting the distribution of the maned wolf (*Chrysocyon brachyurus*) in South America: spatial dynamics and environmental drivers. Mamm Biol 92: 54–61.

[ref18] Consorte-McCrea A (2013) Relationships between the maned wolf and people. In A Consorte-McCrea, E Santos, eds, Ecology and Conservation of the Maned Wolf: Multidisciplinary Perspectives. CRC Press, Boca Raton, pp. 35–52.

[ref19] Constable P, Hinchcliff K, Demma N, Callahan M, Dale B, Fox K, Adams L, Wack R, Kramer L (1998) Electrocardiographic consequences of a peripatetic lifestyle in gray wolves (*Canis lupus*). Comp Biochem Physiol Part A Mol Integr Physiol 120: 557–563. 10.1016/s1095-6433(98)10066-1 9787834

[ref20] Curi NH de A, Coelho CM, Malta M de CC, Magni EMV, Sábato MAL, Araújo AS, Lobato ZIP, Santos JLC, Santos HA, Ragozo AAM et al. (2012) Pathogens of wild maned wolves (*Chrysocyon brachyurus*) in Brazil. J Wildl Dis 48: 1052–1056. 23060508 10.7589/2011-10-304

[ref21] de Souza JC, da Silva RM, Gonçalves MPR, Jardim RJD, Markwith SH (2018) Habitat use, ranching, and human-wildlife conflict within a fragmented landscape in the Pantanal. Brazil Biol Conserv 217: 349–357.

[ref22] Dias WO, Nishimura LT, Cerejo SA, Oliveira LT, Brunelo ATJ, Dias Junior W, Honsho CS, Mattos Junior E, Paulino Junior D (2015) Avaliação do perfil eletrocardiográfico pré e trans-anestesia em lobos-guará [Evaluation of electrocardiographic profile pre and trans-anesthesic in maned wolves]. Arq Bras Med Vet Zootec 1599–1606.

[ref23] Dickens MJ, Delehanty DJ, Michael Romero L (2010) Stress: an inevitable component of animal translocation. Biol Conserv 143: 1329–1341.

[ref24] Dietz JM (1984) Ecology and social organization of the maned wolf (*Chrysocyon brachyurus*). Smithson Contrib Zool 392: 1–51.

[ref25] Diniz LSMM, Lazzarini SM, Angelo MJJ (1999) Problemas médico-veterinários de Lobo-guará (*Chrysocyon brachyurus*) em cativeiro. Rev Educ Contin Med Veterinária Zootec CRMV-SP 2: 34–42.

[ref26] Ditmer MA, Rettler SJ, Fieberg JR, Iaizzo PA, Laske TG, Noyce KV, Garshelis DL (2018a) American black bears perceive the risks of crossing roads. Behav Ecol 29: 667–675.

[ref27] Ditmer MA, Vincent JB, Werden LK, Tanner JC, Laske TG, Iaizzo PA, Garshelis DL, Fieberg JR (2015) Bears show a physiological but limited behavioral response to unmanned aerial vehicles. Curr Biol 25: 2278–2283. 26279232 10.1016/j.cub.2015.07.024

[ref28] Ditmer MA, Werden LK, Tanner JC, Vincent JB, Callahan P, Iaizzo PA, Laske TG, Garshelis DL (2018b) Bears habituate to the repeated exposure of a novel stimulus, unmanned aircraft systems. Conserv Physiol 7. doi: 10.1093/conphys/coy067. PMC6331175 30680216

[ref29] Emmons LH (2012) Maned wolves of Noel Kempff Mercado National Park. Smithson Contrib to Zool 639: 1–35.

[ref30] Estrada AH, Gerlach TJ, Schmidt MK, Siegal-Willott JL, Atkins AL, Van Gilder J, Citino SB, Padilla LR (2009) Cardiac evaluation of clinically healthy captive maned wolves (*Chrysocyon brachyurus*). J Zoo Wildl Med 40: 478–486. 19746863 10.1638/2008-0154.1

[ref31] Estruch JJ, Barken D, Bennett N, Krawiec DK, Ogilvie GK, Powers BE, Polansky BJ, Sueda MT (2020) Evaluation of novel serological markers and autoantibodies in dogs with inflammatory bowel disease. J Vet Intern Med 34: 1177–1186. 32282988 10.1111/jvim.15761 PMC7255684

[ref32] Finkemeier MA, Oesterwind S, Nürnberg G, Puppe B, Langbein J (2019) Assessment of personality types in Nigerian dwarf goats (*Capra hircus*) and cross-context correlations to behavioural and physiological responses. Appl Anim Behav Sci 217: 28–35.

[ref33] Fletchall NB, Zoo JB, Rodden M, Conservation NZ-, Taylor S, Zoo L (1995) Husbandry Manual for the Maned Wolf. Unpublished technical report prepared and distributed by the Association of Zoo and Aquariums Maned Wolf Species Survival Plan.

[ref34] Friard O, Gamba M (2016) BORIS: a free, versatile open-source event-logging software for video/audio coding and live observations. Methods Ecol Evol 7: 1325–1330.

[ref35] Furtado MM, Kashivakura CK, Ferro C, Jácomo AT de A, Silveira L, Astete S (2006) Immobilization of free-ranging maned wolf (*Chrysocyon brachyurus*) with tiletamine and zolazepam in Central Brazil. Source J Zoo Wildl Med 37: 68–70. 10.1638/05-036.1 17312818

[ref36] Gamer M, Lemon J, Fellows I, Singh P (2019) Various Coefficients of Interrater Reliability and Agreement. http://CranR-ProjectOrg/Web/Packages/Irr/IrrPdf 1–32.

[ref37] Heimbürge S, Kanitz E, Otten W (2019) The use of hair cortisol for the assessment of stress in animals. Gen Comp Endocrinol. 270: 10–17. 30287191 10.1016/j.ygcen.2018.09.016

[ref38] Henson LH, Songsasen N, Waddell W, Wolf KN, Emmons L, Gonzalez S, Freeman E, Maldonado J (2017) Characterization of genetic variation and basis of inflammatory bowel disease in the Toll-like receptor 5 gene of the red wolf and the maned wolf. Endanger Species Res 32: 135–144.

[ref39] INPE (2018) Incremento anual de área desmatada no Cerrado Brasileiro—PRODES. *CGOT*. http://www.obt.inpe.br/cerrado (last accessed 13 April 2020).

[ref40] Jachowski DS, Singh NJ (2015) Toward a mechanistic understanding of animal migration: incorporating physiological measurements in the study of animal movement. Conserv Physiol 3. doi: 10.1093/conphys/cov035. PMC4778435 27293720

[ref41] Jachowski DS, Slotow R, Millspaugh JJ (2012) Physiological stress and refuge behavior by African elephants. PLoS One 7. doi: 10.1371/journal.pone.0031818. PMC3284500 22384079

[ref42] Jones MK, Reiter LE, Gilmore MP, Freeman EW, Songsasen N (2018) Physiological impacts of housing maned wolves (*Chrysocyon brachyurus*) with female relatives or unrelated males. Gen Comp Endocrinol 267: 109–115. 29913172 10.1016/j.ygcen.2018.06.007

[ref43] Kleiman DG (1972) Social behavior of the maned wolf (*Chrysocyon brachyurus*) and bush dog (*Speothos venaticus*): a study in contrast. J Mammal 53: 791–806.

[ref44] Kovács L, Kézér FL, Tozsér J, Szenci O, Póti P, Pajor F (2015) Heart rate and heart rate variability in dairy cows with different temperament and behavioural reactivity to humans. PLoS One. 10: e0136294. 26291979 10.1371/journal.pone.0136294 PMC4546236

[ref45] Kozlowska K, Walker P, McLean L, Carrive P (2015) Fear and the defense cascade: clinical implications and management. Harv Rev Psychiatry 23: 263–287. 26062169 10.1097/HRP.0000000000000065 PMC4495877

[ref46] Kreeger TJ, Kuechle VB, Mech LD, Tester JR, Seal US (1990a) Physiological monitoring of gray wolves (*Canis lupus*) by radiotelemetry. J Mammal 71: 258–261.

[ref47] Kreeger TJ, Monson D, Kuechle VB, Seal US, Tester JR (1989) Monitoring heart rate and body temperature in red foxes (*Vulpes vulpes*). Can J Zool 67: 2455–2458.

[ref48] Kreeger TJ, Seal US, Callahan M, Beckel M (1990b) Physiological and behavioral responses of gray wolves (*Canis lupus*) to immobilization with tiletamine and zolazepam. J Wildl Dis 26: 90–94. 2304205 10.7589/0090-3558-26.1.90

[ref49] Larsen RS, Kreeger TJ (2014) Canids. In G West, DJ Heard, N Caulkett, eds, Zoo Animal and Wildlife Immobilization and Anesthesia, EdEd 2. Wiley, Hoboken, pp. 585–598

[ref50] Laske TG, Evans AL, Arnemo JM, Iles TL, Ditmer MA, Fröbert O, Garshelis DL, Iaizzo PA (2018) Development and utilization of implantable cardiac monitors in free-ranging American black and Eurasian brown bears: system evolution and lessons learned. Anim Biotelemetry 6: 13.

[ref51] Laske TG, Garshelis DL, Iaizzo PA (2011) Monitoring the wild black bear’s reaction to human and environmental stressors. BMC Physiol 11: 13. 21849079 10.1186/1472-6793-11-13 PMC3177774

[ref52] Lawrimore JH, Ray R, Applequist S, Korzeniewski B, Menne MJ (2016) Global Summary of the Year (GSOY), Version 1. [1995–2016]. NOAA National Centers for Environmental Information. 10.7289/JWPF-Y430 (last accessed 7 June 2020).

[ref53] Lüdecke D (2020) sjPlot: sjPlot—Data Visualization for Statistics in Social Science. *R Packag version 286*.

[ref54] Lüdecke D, Makowski D, Waggoner P, Patil I (2020) performance: Assessment of Regression Models Performance. *CRAN R Packag*.

[ref55] Lyra-Jorge MC, Ciocheti G, Pivello VR (2008) Carnivore mammals in a fragmented landscape in northeast of São Paulo State. Brazil Biodivers Conserv 17: 1573–1580.

[ref56] Madliger CL, Love OP, Hultine KR, Cooke SJ (2018) The conservation physiology toolbox: status and opportunities. Conserv Physiol 6. doi: 10.1093/conphys/coy029. PMC6007632 29942517

[ref57] Maia OB, Gouveia a MG (2002) Birth and mortality of maned wolves *Chrysocyon brachyurus* (Illiger, 1811) in captivity. Braz J Biol 62: 25–32. 12185920 10.1590/s1519-69842002000100004

[ref58] Massara RL, Maria De Oliveira Paschoal A, Hirsch A, Chiarello AG (2012) Diet and habitat use by maned wolf outside protected areas in eastern Brazil. Trop Conserv Sci 5: 284–300.

[ref59] McCraty R, Atkinson M, Tomasino D, Bradley RT (2009) The coherent heart: heart–brain interactions, psychophysiological coherence, and the emergence of system-wide order. Integr Rev 5: 10–115.

[ref60] McEwen BS, Wingfield JC (2010) What is in a name? Integrating homeostasis, allostasis and stress. Horm Behav 57: 105–111. 19786032 10.1016/j.yhbeh.2009.09.011 PMC2815096

[ref61] Mellor DJ, Hunt S, Gusset M (2015) Caring for Wildlife The World Zoo and Aquarium Animal Welfare Strategy. WAZA Executive Office, Gland, p. 87.

[ref62] Noszczyk-Nowak A, Pasławska U, Nicpoń J (2009) ECG parameters in 24-hour Holter monitoring in healthy dogs. Bull Vet Inst Pulawy 53: 499–502.

[ref63] Padilla LR, Hilton CD (2015) Canidae. In ER Miller, ME Fowler, eds, Fowler’s Zoo and Wild Animal Medicine Vol 8. Saunders, Elsevier, Philadelphia, pp. 457–467.

[ref64] Palme R (2019) Non-invasive measurement of glucocorticoids: advances and problems. Physiol Behav 199: 229–243. 30468744 10.1016/j.physbeh.2018.11.021

[ref65] Paula RC, DeMatteo K (2015) *Chryscocyon brachyurus*. *IUCN Red List Threat Species* 8235. doi:10.2305/IUCN.UK.2015-4.RLTS.T4819A82316878.en.

[ref66] Payne SC, Furness JB, Burns O, Sedo A, Hyakumura T, Shepherd RK, Fallon JB (2019) Anti-inflammatory effects of abdominal vagus nerve stimulation on experimental intestinal inflammation. Front Neurosci 13: 418. 31133776 10.3389/fnins.2019.00418 PMC6517481

[ref67] Perego M, Porteiro Vàzquez DM, Ramera L, Lombardo SF, Pane C, Bontempi LV, Santilli RA (2020) Heart rhythm characterisation during unexplained transient loss of consciousness in dogs. Vet J 263: 105523. 32928492 10.1016/j.tvjl.2020.105523

[ref68] Perini FA, Russo CAM, Schrago CG (2010) The evolution of South American endemic canids: a history of rapid diversification and morphological parallelism. J Evol Biol 23: 311–322. 20002250 10.1111/j.1420-9101.2009.01901.x

[ref69] Péron G, Fleming CH, de Paula RC, Mitchell N, Strohbach M, Leimgruber P, Calabrese JM (2017) Periodic continuous-time movement models uncover behavioral changes of wild canids along anthropization gradients. Ecol Monogr 87: 442–456.

[ref70] Pohlin F, Brabender K, Fluch G, Stalder G, Petit T, Walzer C (2017) Seasonal variations in heart rate variability as an indicator of stress in free-ranging pregnant Przewalski’s horses (*E. ferus przewalskii*) within the Hortobágy National Park in Hungary. Front Physiol 8: 664. 28936179 10.3389/fphys.2017.00664 PMC5594093

[ref71] Queirolo D, Moreira JR, Soler L, Emmons LH, Rodrigues FHG, Pautasso AA, Cartes JL, Salvatori V (2011) Historical and current range of the near threatened maned wolf *Chrysocyon brachyurus* in South America. Oryx 45: 296–303.

[ref72] R Core Team (2019) R: A Language and Environment for Statistical Computing. In R Found Stat Comput Vienna, Austria. https://www.r-project.org/.

[ref73] Rao R, Androulakis IP (2019) The physiological significance of the circadian dynamics of the HPA axis: interplay between circadian rhythms, allostasis and stress resilience. Horm Behav 110: 77–89. 30862458 10.1016/j.yhbeh.2019.02.018

[ref74] Silva FAM, Assad ED, Steinke ET, Müller AG (2008) Clima do bioma cerrado. In ACS Albuquerque, AG Silva, eds, Agricultura Tropical: Quatro Décadas de Inovações Tecnológicas, Institucionais e Políticas. Brasília, DF, Embrapa Informação Tecnológica, pp. 93–148.

[ref75] Songsasen N, Rodden MD (2010) The role of the species survival plan in maned wolf *Chrysocyon brachyurus* conservation. Int Zoo Yearb 44: 136–148.

[ref76] Spercoski KM, Morais RN, Morato RG, de Paula RC, Azevedo FC, May-Júnior JA, Santos JP, Reghelin AL, Wildt DE, Songsasen N (2012) Adrenal activity in maned wolves is higher on farmlands and park boundaries than within protected areas. Gen Comp Endocrinol 179: 232–240. 22917914 10.1016/j.ygcen.2012.08.002

[ref77] Stemmler G, Aue T, Wacker J (2007) Anger and fear: separable effects of emotion and motivational direction on somatovisceral responses. Int J Psychophysiol 66: 141–153. 17544534 10.1016/j.ijpsycho.2007.03.019

[ref78] Støen O-G, Ordiz A, Evans AL, Laske TG, Kindberg J, Fröbert O, Swenson JE, Arnemo JM (2015) Physiological evidence for a human-induced landscape of fear in brown bears (*Ursus arctos*). Physiol Behav 152: 244–248. 26476156 10.1016/j.physbeh.2015.09.030

[ref79] Taylor CR, Karas RH, Weibel ER, Hoppeler H (1987) Adaptive variation in the mammalian respiratory system in relation to energetic demand: II. Reaching the limits to oxygen flow. Respir Physiol 69: 7–26. 10.1016/0034-5687(87)90097-1 3616184

[ref86] Vieira-da-Motta O, Eckhardt-de-Pontes LA, Petrucci MP, dos Santos IP, da Cunha IC, Morato RG (2014) Microbiota and anthropic interference on antimicrobial resistance profile of bacteria isolated from Brazilian Maned-wolf (Chrysocyon brachyurus). Braz J Microbiol 44: 1321–1326. 24688529 10.1590/s1517-83822013000400042 PMC3958205

[ref80] Viola AU, Simon C, Ehrhart J, Geny B, Piquard F, Muzet A, Brandenberger G (2002) Sleep processes exert a predominant influence on the 24-h profile of heart rate variability. J Biol Rhythms 17: 539–547. 12465887 10.1177/0748730402238236

[ref81] Vynne C, Booth RK, Wasser SK (2014) Physiological implications of landscape use by free-ranging maned wolves ( *Chrysocyon brachyurus* ) in Brazil. J Mammal 95: 696–706.

[ref82] Vynne C, Keim JL, Machado RB, Marinho-Filho J, Silveira L, Groom MJ, Wasser SK (2011) Resource selection and its implications for wide-ranging mammals of the Brazilian Cerrado. PLoS One 6. doi: 10.1371/journal.pone.0028939. PMC3243687 22205984

[ref83] Yerga J, Calzada J, Manteca X, Vargas A, Pérez MJ, Palomares F, Rivas A (2015) Ontogeny of daily activity and circadian rhythm in the Iberian lynx (*Lynx pardinus*). Appl Anim Behav Sci 169: 62–68.

[ref84] Zanette LY, Hobbs EC, Witterick LE, MacDougall-Shackleton SA, Clinchy M (2019) Predator-induced fear causes PTSD-like changes in the brains and behaviour of wild animals. Sci Rep 9: 1–10. 31391473 10.1038/s41598-019-47684-6 PMC6685979

