# Supplementary material for: Inside out: heart rate monitoring to advance the welfare and conservation of maned wolves (Chrysocyon brachyurus)
Source: Conserv Physiol. 2021 Jun 24;9(1):coab044. doi: 10.1093/conphys/coab044 (PMC8224209; doi:10.1093/conphys/coab044)
Supplement: suppl_data_coab044 [file suppl_data_coab044.zip › Supplementary material SM1 review 2.docx]

**Table 2**. Housing information about maned wolves implanted with a biologger (Reveal LINQ™, Medtronic Inc., MN, USA) for a heart monitoring study at the Smithsonian Conservation Biology Institute, Front Royal, VA, from 2018 to 2019.

| Subject | Housing Status | Onset |
| --- | --- | --- |
| Female 1 | Family group (Male 1 and 2 pups) | Feb-2018 |
|  | Single next to Male 1 | Oct-2018 |
| Female 2 | Paired with her female offspring | May-2017 |
|  | Single next to Male 2 | Jul-2018 |
|  | Paired with Male 2 | Oct-2018 |
| Female 3 | Single next to Male 3 | Jun-2018 |
|  | Single next to an adult male | Mar-2019 |
| Male 1 | Family group (Female 1 and 2 cubs) | Feb-2018 |
|  | Family group (2 cubs) | Oct-2018 |
|  | Single next to Female 1 | Nov-2018 |
| Male 2 | Family group (1 adult female + 4 cubs) | Dec-2017 |
|  | Single next to Female 2 | Jul-2018 |
|  | Paired with Female 2 | Oct-2018 |
| Male 3 | Single next to Female 3 | Jun-2018 |
|  | Single next to two females (Female 3 and 1 adult female) | Jul-2018 |
|  | Single next to two females | Nov-18 |
|  |  |  |

**Table 3**. Description of mixed models fit for data analysis, using the *lme4* R-Package. Linear mixed models (l*mer*) were estimated using restricted maximum likelihood (REML) and nloptwrap optimizer. The logistic mixed model (glmer; model 6) was estimated using maximum likelihood, Nelder-Mead optimizer and a binomial error distribution.

| Model | Response variable | Fixed effects | Random effects | Formula | Test |
| --- | --- | --- | --- | --- | --- |
| 1 | HR daily average | Null | Subject | lmer (HR ~ 1 + (1\|Subject)) | Differences between individuals |
| 2 | HRV daily average | Null | Subject | lmer (HRV ~ 1 + (1\|Subject)) | Differences between individuals |
| 3 | HR daily average | Sex | Subject | lmer (HR ~ Sex + (1\|Subject)) | Differences between sex |
| 4 | HRV daily average | Sex | Subject | lmer (HRV ~ Sex + (1\|Subject)) | Differences between sex |
| 5 | HR average per light period | Light period, Rearing Method | Subject, Date | lmer (HR ~ Light*Rearing + (1\|Subject/Date)) | Effects of light period (day, dusk, night, dawn) and rearing method (parent, hand) on HR |
| 6 | Proportion of time active per light period | Light period, Rearing Method | Subject, Date | glmer (cbind(Time active, Time inactive) ~ Light*Rearing + (1\|Subject/Date)) | Effects of light period and rearing method on proportion of time active |
| 7 | Two-min HR averages per restraint period | Restraint period, Rearing method | Subject | lmer (HR ~ Restraint*Rearing + (1\|Subject)) | Effects of restraint period (baseline, pre, in-crate, post) and Rearing method on HR average during restraint events for data download |
| 8 | Two-min HR averages during In-crate period of restraint | Behavior, Rearing Method, Number of Previous restraints | Subject | lmer (HR ~ Behavior*Rearing + Number of previous restraints + (1\|Subject)) | Effects of apparent behavior (calm or stressed), rearing method (hand or parent), and number of previous restraint events on HR average during time held in a squeeze crate (~14 min). |

**Table 4**. Daily time budget for implant-specific behaviors (observing, licking, or gnawing at the implant site) in six maned wolves observed daily for 10 min during the first 15 days after being implanted with a biologger (Reveal LINQ™, Medtronic Inc., MN, USA). Percentages are relative to animals’ visible time per day.

| Subject | Time engaged in Implant-Specific Behaviors | | Daily Visible Time |
| --- | --- | --- | --- |
|  | Average  sec (%) | Maximum  sec (%) | Average  (sec) |
| Female 1 | 57 (9.8) | 315 (52.4) | 547 |
| Female 2 | 1 (0.1) | 3 (0.4) | 563 |
| Female 3 | 16 (6.2) | 65 (36.0) | 297 |
| Male 1 | 7 (1.5) | 29 (6.2) | 382 |
| Male 2 | 12 (2.6) | 90 (15.4) | 536 |
| Male 3 | 5 (0.9) | 14 (2.6) | 345 |

**MODEL 1. Random effect of *Subject* on daily HR average (bpm).** Constant (intercept-only) linear mixed model (estimated using REML and nloptwrap optimizer) to predict daily HR average (formula: HR ~ 1). The model included *Subject* as random effect (formula: ~1 | Subject). 95% Confidence Intervals (CIs) and p-values were computed using the Wald approximation.

|  | **Effect of Subject on Maned wolf Daily HR Average** | | | | |
| --- | --- | --- | --- | --- | --- |
| *Predictors* | *Estimates* | *CI* | *Statistic* | *p* | *df* |
| (Intercept) | 89.65 | 78.78 – 100.53 | 21.19 | **<0.001** | 5.00 |
| **Random Effects** | | | | | |
| σ^2^ | 82.47 | | | | |
| τ_00_ _Subject_ | 107.13 | | | | |
| ICC | 0.57 | | | | |
| N _Subject_ | 6 | | | | |
| Observations | 2048 | | | | |
| Marginal R^2^ / Conditional R^2^ | 0.000 / 0.565 | | | | |

**MODEL 2. Random effect of *Subject* on daily HRV (ms; SDANN).** Constant (intercept-only) linear mixed model (estimated using REML and nloptwrap optimizer) to predict daily HRV (formula: HRV ~ 1). The model included Subject as random effect (formula: ~1 | Subject). 95% Confidence Intervals (CIs) and p-values were computed using the Wald approximation.

|  | **Random Effect of Subject on Maned wolf HRV (SDANN)** | | | |
| --- | --- | --- | --- | --- |
| *Predictors* | *Estimates* | *CI* | *Statistic* | *p* |
| (Intercept) | 190.93 | 149.61 – 232.25 | 9.06 | **<0.001** |
| **Random Effects** | | | | |
| σ^2^ | 1724.20 | | | |
| τ_00_ _Subject_ | 2661.75 | | | |
| ICC | 0.61 | | | |
| N _Subject_ | 6 | | | |
| Observations | 1973 | | | |
| Marginal R^2^ / Conditional R^2^ | 0.000 / 0.607 | | | |

**MODEL 3. Effect of *Sex* on daily HR average (bpm).** Linear mixed model (estimated using REML and nloptwrap optimizer) to predict daily HR average with *Sex* (formula: HR ~ Sex). The model included *Subject* as random effect (formula: ~1 | Subject). The model's total explanatory power is substantial (conditional R2 = 0.60) and the part related to the fixed effects alone (marginal R2) is of 0.10. 95% Confidence Intervals (CIs) and p-values were computed using the Wald approximation.

|  | **Effect of Sex on Maned wolf Daily HR Average** | | | | |
| --- | --- | --- | --- | --- | --- |
| *Predictors* | *Estimates* | *CI* | *Statistic* | *p* | *df* |
| (Intercept) | 85.16 | 73.62 – 96.70 | 14.47 | **<0.001** | 2044.00 |
| Male | 8.98 | -7.34 – 25.30 | 1.08 | 0.281 | 2044.00 |
| **Random Effects** | | | | | |
| σ^2^ | 82.47 | | | | |
| τ_00_ _Subject_ | 103.71 | | | | |
| ICC | 0.56 | | | | |
| N _Subject_ | 6 | | | | |
| Observations | 2048 | | | | |
| Marginal R^2^ / Conditional R^2^ | 0.098 / 0.600 | | | | |

**MODEL 4. Effect of *Sex* on HRV (ms; SDANN).** Linear mixed model (estimated using REML and nloptwrap optimizer) to predict daily HRV with *Sex* (formula: HRV ~ Sex). The model included *Subject* as random effect (formula: ~1 | Subject). The model's total explanatory power is substantial (conditional R2 = 0.61) and the part related to the fixed effects alone (marginal R2) is of 0.31.

|  | **Effect of Sex on Maned wolf Daily HRV (SDANN)** | | | | |
| --- | --- | --- | --- | --- | --- |
| *Predictors* | *Estimates* | *CI* | *Statistic* | *p* | *df* |
| (Intercept) | 227.85 | 187.20 – 268.49 | 10.99 | **<0.001** | 1969.00 |
| Male | -73.84 | -131.31 – -16.36 | -2.52 | **0.012** | 1969.00 |
| **Random Effects** | | | | | |
| σ^2^ | 1724.20 | | | | |
| τ_00_ _Subject_ | 1284.52 | | | | |
| ICC | 0.43 | | | | |
| N _Subject_ | 6 | | | | |
| Observations | 1973 | | | | |
| Marginal R^2^ / Conditional R^2^ | 0.312 / 0.606 | | | | |

**MODEL 5. Effect of *Light* and *Rearing Method* on HR (bpm).** Linear mixed model (estimated using REML and nloptwrap optimizer) to predict HR average with *Light* and *Rearing.Method* (formula: HR ~ Light * Rearing.Method). The model included *Date* and *Subject* as random effects (formula: list(~1 | Date:Subject, ~1 | Subject)). The model's total explanatory power is substantial (conditional R2 = 0.47) and the part related to the fixed effects alone (marginal R2) is of 0.12. 95% Confidence Intervals (CIs) and p-values were computed using the Wald approximation.

|  | **Effects of Light and Rearing Method on Maned wolf HR** | | | | |
| --- | --- | --- | --- | --- | --- |
| *Predictors* | *Estimates* | *CI* | *Statistic* | *p* | *df* |
| (Intercept) | 80.57 | 68.86 – 92.28 | 13.48 | **<0.001** | 7029.00 |
| Dusk | 22.24 | 20.93 – 23.54 | 33.37 | **<0.001** | 7029.00 |
| Night | 11.26 | 9.96 – 12.57 | 16.92 | **<0.001** | 7029.00 |
| Dawn | 5.86 | 4.56 – 7.17 | 8.81 | **<0.001** | 7029.00 |
| Hand-reared | 15.10 | -5.19 – 35.38 | 1.46 | 0.145 | 7029.00 |
| Dusk*Hand-reared | -15.58 | -17.88 – -13.28 | -13.29 | **<0.001** | 7029.00 |
| Night*Hand-reared | -9.65 | -11.94 – -7.35 | -8.24 | **<0.001** | 7029.00 |
| Dawn*Hand-reared | -15.28 | -17.57 – -12.98 | -13.05 | **<0.001** | 7029.00 |
| **Random Effects** | | | | | |
| σ^2^ | 263.00 | | | | |
| τ_00_ _Date:Subject_ | 30.85 | | | | |
| τ_00_ _Subject_ | 141.81 | | | | |
| ICC | 0.40 | | | | |
| N _Date_ | 364 | | | | |
| N _Subject_ | 6 | | | | |
| Observations | 7040 | | | | |
| Marginal R^2^ / Conditional R^2^ | 0.12 / 0.47 | | | | |

**MODEL 6. Effect of *Light* and *Rearing Method* on proportion of time active (Minutes active/Minutes Inactive).** Logistic mixed model (estimated using ML and Nelder-Mead optimizer) to predict proportion of time active with *Light* and *Rearing.Method* (formula: cbind(Time Active, Time Inactive) ~ Light * Rearing.Method). The model included *Date* and *Subject* as random effects (formula: list(~1 | Date:Subject, ~1 | Subject). The model's total explanatory power is moderate (conditional R2 = 0.23) and the part related to the fixed effects alone (marginal R2) is of 0.12. Error distribution: binomial. 95% Confidence Intervals (CIs) and p-values were computed using the Wald approximation.

|  | **Effects of Light and Rearing Method on Maned wolf Activity** | | | |
| --- | --- | --- | --- | --- |
| *Predictors* | *Odds Ratios* | *CI* | *Statistic* | *p* |
| (Intercept) | 0.18 | 0.14 – 0.25 | -10.79 | **<0.001** |
| Dusk | 8.00 | 7.87 – 8.14 | 245.02 | **<0.001** |
| Night | 4.27 | 4.23 – 4.30 | 363.70 | **<0.001** |
| Dawn | 3.41 | 3.35 – 3.46 | 145.34 | **<0.001** |
| Hand-reared | 1.51 | 0.89 – 2.57 | 1.53 | 0.127 |
| Dusk *Hand-reared | 0.27 | 0.26 – 0.28 | -88.98 | **<0.001** |
| Night *Hand-reared | 0.35 | 0.35 – 0.36 | -153.82 | **<0.001** |
| Dawn *Hand-reared | 0.23 | 0.22 – 0.24 | -88.09 | **<0.001** |
| **Random Effects** | | | | |
| σ^2^ | 3.29 | | | |
| τ_00_ _Date:Subject_ | 0.38 | | | |
| τ_00_ _Subject_ | 0.10 | | | |
| ICC | 0.13 | | | |
| N _Date_ | 364 | | | |
| N _Subject_ | 6 | | | |
| Observations | 7040 | | | |
| Marginal R^2^ / Conditional R^2^ | 0.12 / 0.23 | | | |

**MODEL 7. Effects of *Restraint* periods and *Rearing Method* on 2-min HR averages (bpm).** Linear mixed model (estimated using REML and nloptwrap optimizer) to predict 2-min HR averages with restraint period and rearing method (formula: HR ~ Restraint * Rearing). The model included subject as random effect (formula: ~1 | Subject). The model's total explanatory power is substantial (conditional R2 = 0.52) and the part related to the fixed effects alone (marginal R2) is of 0.33. 95% Confidence Intervals (CIs) and p-values were computed using the Wald approximation.

|  | **Effects of Restraint in a Crate and Rearing Method on HR (bpm)** | | | | |
| --- | --- | --- | --- | --- | --- |
| *Predictors* | *Estimates* | *CI* | *Statistic* | *p* | *df* |
| (Intercept) | 87.67 | 71.10 – 104.24 | 10.37 | **<0.001** | 1214.00 |
| Pre-Crate | 10.33 | 5.30 – 15.37 | 4.02 | **<0.001** | 1214.00 |
| In-Crate | 79.83 | 73.65 – 86.01 | 25.32 | **<0.001** | 1214.00 |
| Post-Crate | 27.23 | 22.54 – 31.91 | 11.39 | **<0.001** | 1214.00 |
| Hand-reared | 14.65 | -14.46 – 43.76 | 0.99 | 0.324 | 1214.00 |
| Pre-Crate*Hand-reared | -5.32 | -16.01 – 5.36 | -0.98 | 0.329 | 1214.00 |
| In-Crate*Hand-reared | -41.12 | -54.22 – -28.01 | -6.15 | **<0.001** | 1214.00 |
| Post-Crate*Hand-reared | -6.31 | -16.25 – 3.63 | -1.24 | 0.213 | 1214.00 |
| **Random Effects** | | | | | |
| σ^2^ | 644.32 | | | | |
| τ_00_ _Animal_ | 268.93 | | | | |
| ICC | 0.29 | | | | |
| N _Animal_ | 6 | | | | |
| Observations | 1224 | | | | |
| Marginal R^2^ / Conditional R^2^ | 0.325 / 0.524 | | | | |

**Model 8. Effects of apparent *Behavior*, *Rearing Method*, and *Number of Previous Restraints* on 2-min HR averages (bpm) during the time held in a crate (~ 14 min).** Linear mixed model (estimated using REML and nloptwrap optimizer) to predict 2-min HR averages when *In Crate* with apparent behavior, rearing method, and number of previous restraint events (formula: HR ~ Behavior*Rearing + Previous restraints). The model included subject as random effect (formula: ~1 | Subject). The model's total explanatory power is substantial (conditional R2 = 0.52) and the part related to the fixed effects alone (marginal R2) is of 0.25. 95% Confidence Intervals (CIs) and p-values were computed using the Wald approximation.

|  | **Effects of Apparent Behavior, Rearing Method and Number of Previous Restraints on HR *In Crate*** | | | | |
| --- | --- | --- | --- | --- | --- |
| *Predictors* | *Estimates* | *CI* | *Statistic* | *p* | *df* |
| (Intercept) | 164.07 | 149.65 – 178.49 | 22.30 | **<0.001** | 145.00 |
| Stressed | 6.60 | -1.62 – 14.82 | 1.57 | 0.115 | 145.00 |
| Hand-reared | -14.29 | -37.41 – 8.83 | -1.21 | 0.226 | 145.00 |
| Previous restraints | -0.01 | -2.46 – 2.43 | -0.01 | 0.992 | 145.00 |
| Stressed*Hand-reared | -20.10 | -33.14 – -7.06 | -3.02 | **0.003** | 145.00 |
| **Random Effects** | | | | | |
| σ^2^ | 274.92 | | | | |
| τ_00_ _Animal_ | 155.35 | | | | |
| ICC | 0.36 | | | | |
| N _Animal_ | 6 | | | | |
| Observations | 152 | | | | |
| Marginal R^2^ / Conditional R^2^ | 0.250 / 0.521 | | | | |
