# Supplementary material for: Inside out: heart rate monitoring to advance the welfare and conservation of maned wolves (Chrysocyon brachyurus)
Source: Conserv Physiol. 2021 Jun 24;9(1):coab044. doi: 10.1093/conphys/coab044 (PMC8224209; doi:10.1093/conphys/coab044)
Supplement: suppl_data_coab044 [file suppl_data_coab044.zip › Supplementary material SM2.docx]

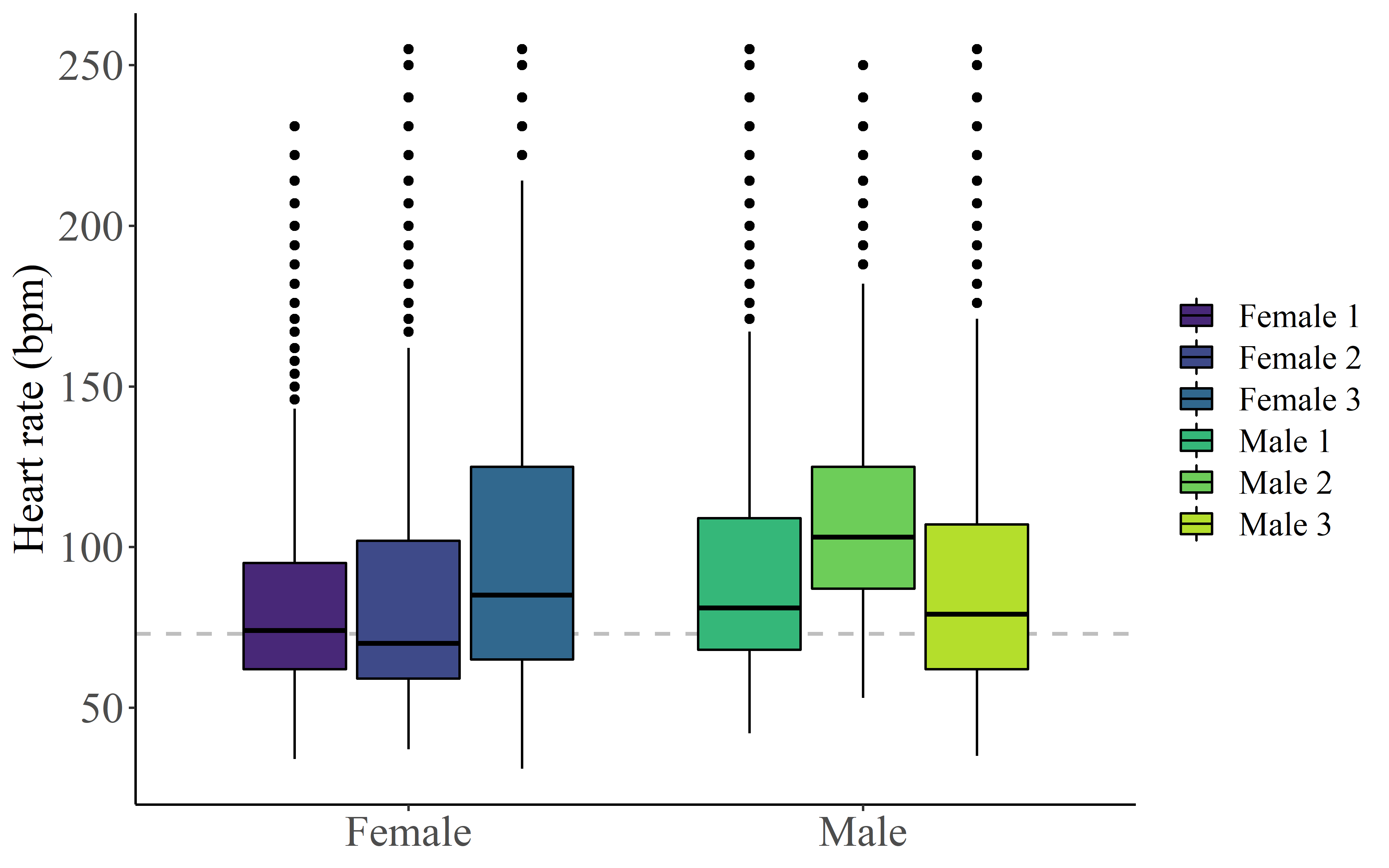


**Figure 1**. Heart rate (HR) average every 2 min (bpm) in 6 captive maned wolves implanted with a biologger (Reveal LINQ™, Medtronic Inc., MN, USA) at the Smithsonian Conservation Biology Institute, Front Royal, VA, USA. Data collected from June 2018 to August 2019. The horizontal dashed line shows the overall resting HR for all individuals during the one-year study period (73 bpm).


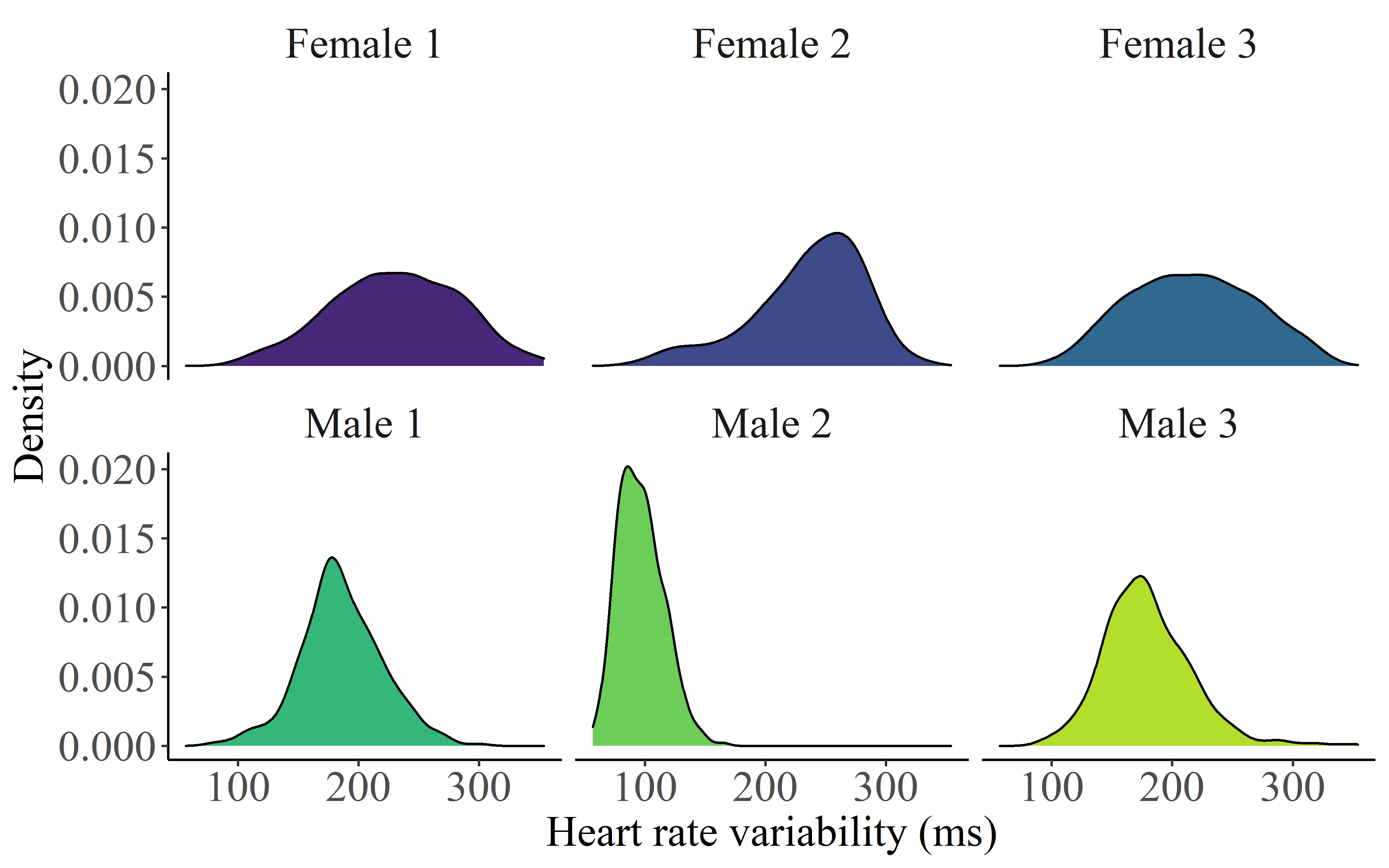


**Figure 2**. Daily individual heart rate variability (standard deviation of beat-to-beat intervals in sinus rhythm using 5-min medians of all R-R intervals in a 24 -h recording; SDANN, ms) density plot in 6 captive maned wolves implanted with a biologger (Reveal LINQ™, Medtronic Inc., MN, USA) at the Smithsonian Conservation Biology Institute, Front Royal, VA, USA. Data collected from June 2018 to August 2019.
